# Susceptibility to cellular stress in PS1 mutant N2a cells is associated with mitochondrial defects and altered calcium homeostasis

**DOI:** 10.1038/s41598-020-63254-7

**Published:** 2020-04-15

**Authors:** Liliana Rojas-Charry, Sergio Calero-Martinez, Claudia Morganti, Giampaolo Morciano, Kyungeun Park, Christian Hagel, Stefan J. Marciniak, Markus Glatzel, Paolo Pinton, Diego Sepulveda-Falla

**Affiliations:** 1 Institute of Neuropathology, University Medical Center Hamburg-Eppendorf, Hamburg, Germany; 2 Department of Morphology, Surgery and Experimental Medicine, Section of Pathology, Oncology and Experimental Biology, University of Ferrara, 44121 Ferrara, Italy; 3 Cambridge Institute for Medical Research (CIMR), University of Cambridge, Cambridge, UK

**Keywords:** Alzheimer's disease, Stress and resilience

## Abstract

Presenilin 1 (PS1) mutations are the most common cause of familial Alzheimer’s disease (FAD). PS1 also plays a role in cellular processes such as calcium homeostasis and autophagy. We hypothesized that mutant presenilins increase cellular vulnerability to stress. We stably expressed human PS1, mutant PS1E280A and mutant PS1Δ9 in mouse neuroblastoma N2a cells. We examined early signs of stress in different conditions: endoplasmic reticulum (ER) stress, calcium overload, oxidative stress, and Aβ 1–42 oligomers toxicity. Additionally, we induced autophagy via serum starvation. PS1 mutations did not have an effect in ER stress but PS1E280A mutation affected autophagy. PS1 overexpression influenced calcium homeostasis and generated mitochondrial calcium overload modifying mitochondrial function. However, the opening of the mitochondrial permeability transition pore (MPTP) was affected in PS1 mutants, being accelerated in PS1E280A and inhibited in PS1Δ9 cells. Altered autophagy in PS1E280A cells was neither modified by inhibition of γ-secretase, nor by ER calcium retention. MPTP opening was directly regulated by γ-secretase inhibitors independent on organelle calcium modulation, suggesting a novel direct role for PS1 and γ-secretase in mitochondrial stress. We identified intrinsic cellular vulnerability to stress in PS1 mutants associated simultaneously with both, autophagic and mitochondrial function, independent of Aβ pathology.

## Introduction

Alzheimer Disease (AD) is the most common form of dementia, mainly attributed to altered processing and deposition of extracellular Aβ plaques and intracellular neurofibrillary tangles in the brain^1^. Current understanding of AD pathophysiology indicates impairment of several cellular processes such as lipid metabolism, mitochondrial function and autophagy, leading eventually to cellular stress and death. A multifactorial model for AD proposes a cellular phase in which Amyloid beta (Aβ) pathology drives Tau hyperphosphorylation inducing cellular damage^2^.

Amyloid Precursor Protein (APP), Presenilin 1 (PS1) and Presenilin 2 (PS2) autosomal dominant mutations are causative of familial AD (FAD)^3^. FAD is characterized by its severity and earlier disease onset, together with severe brain atrophy indicating increased neuronal death^4^. Presenilins are the catalytic component of the γ-secretase complex, playing a role in Aβ generation. The pathological severity of FAD suggests a direct neurodegenerative role of PS1 mutations, whether by increased production of toxic Aβ or by other mechanisms^5^. Nevertheless, PS1 has also been related to other cellular functions, such as protein trafficking, Wnt/β-catenin signaling, apoptosis and the disruption of calcium homeostasis^6–8^. Accordingly, PS1 mutations have been associated to increased cellular stress or death responses such as endoplasmic reticulum (ER) stress^9^, oxidative stress^10,11^, autophagy^12^, and apoptosis^13^. Abnormal calcium homeostasis and its pathological role (calcium overload) in AD have attracted attention during recent years. Calcium signaling is involved in different pathways, being essential for synaptic mechanisms, protein folding processes, cell survival and death, among many others^14^. Regarding FAD, PS1 and PS2 have been associated with altered calcium signaling and PS1 has been found to affect calcium dynamics in lysosomes and ER^8,14^. Those changes in neuronal and synaptic calcium could lead to synaptic and neuronal toxicity^14,15^.

There is a large population carrying a single PS1 mutation, E280A, with ~6,000 individuals and ~600 affected carriers^16^. The PS1E280A mutation is localized in exon 8 of the PSEN1 gene and substitutes a glutamic acid for an alanine in the loop region of PS1^16^. It affects APP processing and Aβ generation^17^. Also, this mutation may affect the processing of other γ-secretase substrates^18,19^. On the other hand, mitochondrial dysfunction has been identified as one key process in AD pathophysiology. Altered processes include oxidative stress, mitochondrial dynamics and calcium dysregulation^20–22^. Our previous studies in brain tissue of PS1 E280A FAD patients and cellular models for PS1E280A mutation showed altered mitochondrial function and evidence of altered calcium homeostasis, associated with increased Purkinje cells loss and cerebellar damage^23^. These findings can indicate an increased cellular vulnerability to stress in PS1 mutant cells, induced by these mechanisms. Finally, it has been suggested that mitochondrial calcium dysregulation in AD can result from abnormal aperture of the mitochondrial permeability transition pore (MPTP) due to a modulatory effect of Aβ^24^. In the present study, we analyzed different cellular stress pathways in N2a cells overexpressing wild type human PS1 (hPS1WT), PS1 E280A (hPS1E280A) and PS1 exon 9 deletion (hPS1Δ9) mutations. We found that although PS1 overexpression has an impact in mitochondrial function, only PS1 mutations confer further vulnerability to autophagy and mitochondrial stress responses on a mutation-specific manner seemingly by γ-secretase dependent and independent mechanisms, including direct MPTP regulation.

## Results

### Establishment of a cellular stress model in N2a cells

**Figure 1:**
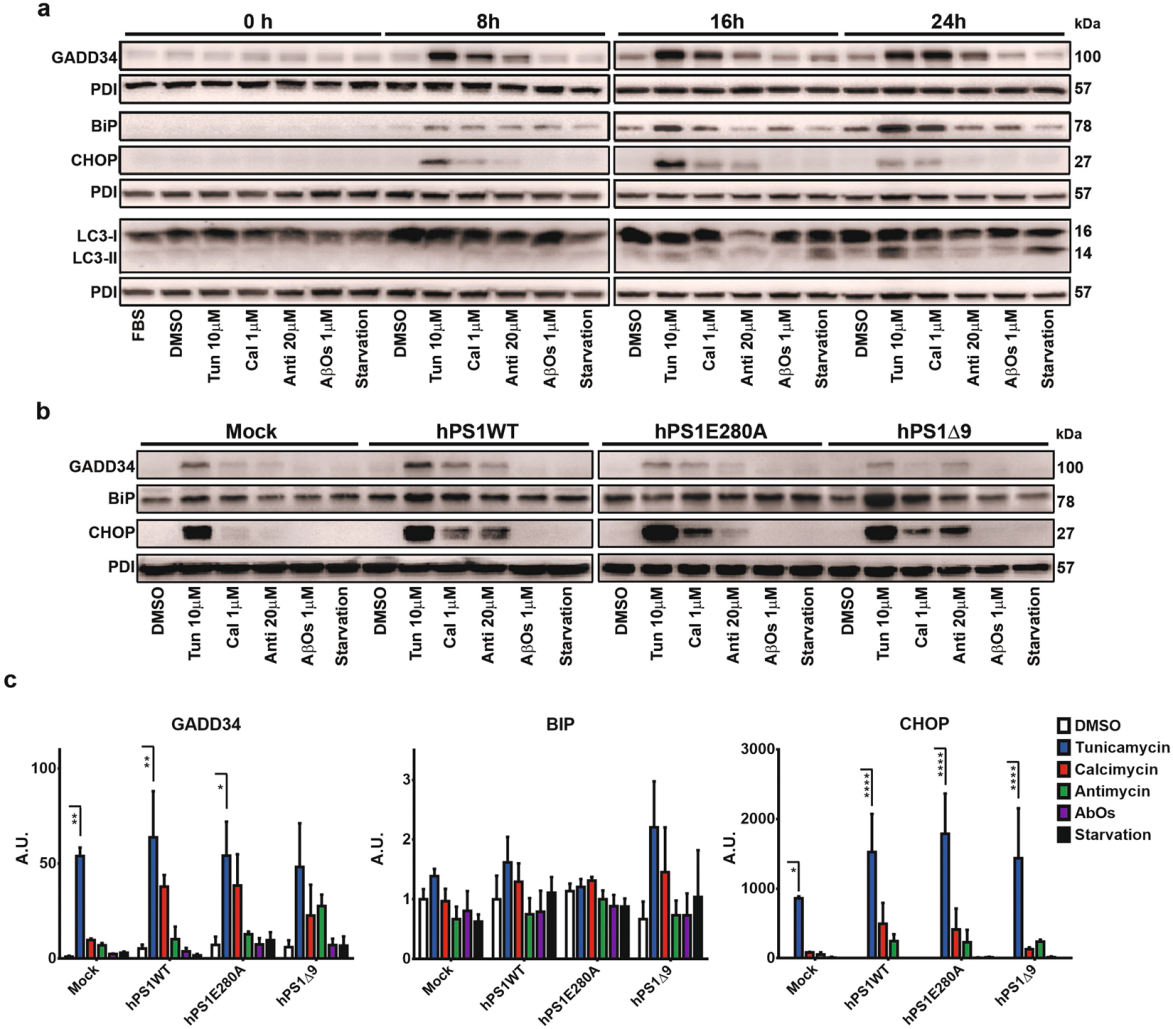
PS1 mutations do not affect ER stress response. (**a**) N2a mock transfected cells were treated with FBS, DMSO, tunicamycin 10 µM, calcimycin 1 µM, antimicyn 20 µM, Aβ 1–42 oligomers (AβOs) 1 µM, and serum starvation during 8, 16, and 24 h. Representative western blots for ER stress and autophagy markers are depicted. PDI was used as loading control. (**b**) N2a mock and PS1 stably transfected cells were treated with FBS, DMSO, tunicamycin 10 µM, calcimycin 1 µM, antimicyn 20 µM, Aβ 1–42 oligomers (AβOs) 1 µM, and serum starvation during 16 h. Representative western blots of protein steady state levels of GADD34, BiP, and CHOP in N2a cells overexpressing hPS1, hPS1-E280A or hPS1Δ9 are depicted. (**c**) Densitometric analysis of GADD34, BiP, and CHOP steady state levels. Mean ± SEM is presented for all experiments, Two-Way ANOVA. *p < 0.05,**p < 0.01, ***p < 0.001,****p < 0.0001, n = 3.

First, we confirmed hPS1 overexpression in transfected cells using western blot and qPCR (Fig. S1A–C). We checked functional effects of hPS1 overexpression, showing increased APP production without modifying Aβ 1–40 or Aβ 1–42 production (Fig. S1D,E). As expected, overexpression of hPS1E280A increased Aβ 1–40 or Aβ 1–42 levels (Fig. S1E). Finally, we observed that hPS1 overexpression did not modify significantly the subcellular distribution of PS1 (Fig. S2A,B). For our cellular stress model, we selected tunicamycin as an ER stress inducer and serum starvation as an autophagy inducer. Given PS1 role in calcium homeostasis, we treated cells with calcimycin to see the effect of calcium overload in ER stress and autophagy. Also, we evaluated antimycin as a mitochondrial stressor given possible mitochondrial stress crosstalk with ER stress and autophagy^25^. Finally, we assessed the effect of Aβ 1–42 (Aβ 1–42) as a positive control for putative mechanisms of stress in AD. To define the optimal time and dosage for each treatment, we incubated wild type N2a cells with each stressor or in the absence of FBS (for serum starvation) and extracted total protein after 0 h, 8 h, 16 h and 24 h. After 8 h of treatment we could identify that tunicamycin, calcimycin and antimycin already elicited ER stress in wild type N2a cells as evidenced by the induction of BiP, CHOP and GADD34. ER stress remained at 16 h and 24 h. Also, at 16 h some degree of LC3B-II conversion was seen with all treatments, with serum starvation depicting more (Fig. 1a). At 24 h, starvation showed clear LC3B-II conversion and some crosstalk between pathways became evident with tunicamycin leading to higher LC3B-II conversion. Additionally, N2a cells can start to differentiate under FBS starvation conditions at 24 hours. Consequently, we selected 16 h as the optimal time for inducing ER stress and autophagy in our cellular model.

### PS1 mutations do not modify ER stress in N2a cells

We aimed to assess if PS1E280A mutation affects mechanisms of cellular stress, with ER stress and autophagy as the first targets given that alterations in both processes have previously been associated with PS1 mutations^9,12^. As expected, tunicamycin induced markers of ER stress (BiP, CHOP and GADD34) in mock, hPS1WT and hPS1E280A cells. However, in hPS1Δ9 cells only CHOP was reproducibly induced by tunicamycin (Fig. 1b,c). Previously, varied, sometimes contradictory effects of PS1 on ER stress signaling have been published using diverse markers. Reported findings are inconsistent regarding whether ER stress response is enhanced or dysfunctional due to mutations in PS1 (for references see Supplementary Table 1). This might reflect the use of differing cellular models or strategies involving modified PS1. To shed light on this, we silenced murine PS1 expression in N2a cells (PS1KD) using siRNA targeting exon 3 of the PSEN1 gene and treated them with tunicamycin to generate ER stress on a timeline up to 16 hours. Although transfection of murine PS1 and control siRNA could have affected basal levels of GADD34 and BiP, tunicamycin treatment elicited increased levels of BiP after 8 h, and of GADD34 after 8 h and 16 h of treatment in both groups without visible differences between PS1KD or control cells (Fig. S3A,B). Additionally, we evaluated whether the total absence of Presenilins affected the induction of ER stress. For that we used MEFs generated from Presenilin double knock out mice (PS KO−/−)^26^. By western blot, we observed some differences in the activation of BIP and GADD34. PS KO−/− cells showed a slight delayed activation of both proteins (Fig. S3C). Next, we evaluated ER stress markers XBP1 and CHOP by RT-PCR. Only CHOP showed some difference with increased levels by tunicamycin treatment after 8 hours in PS KO−/− cells (Fig. S3D). Finally, we evaluated GADD34, BiP, and phosphorylated eIF2α steady state levels with western blot in WT cells and PS1 KO−/− cells, with and without stable rescue for hPS1WT and hPS1E280A (Fig. S3E). We found no statistically significant differences within cell lines for ER stress markers (Fig. S3F). Taken together, these results indicate that in N2a and MEF cells, PS1 mutations or downregulation do not affect ER stress responses.

### Autophagic system impairment in hPS1 E280A N2a cells

**Figure 2:**
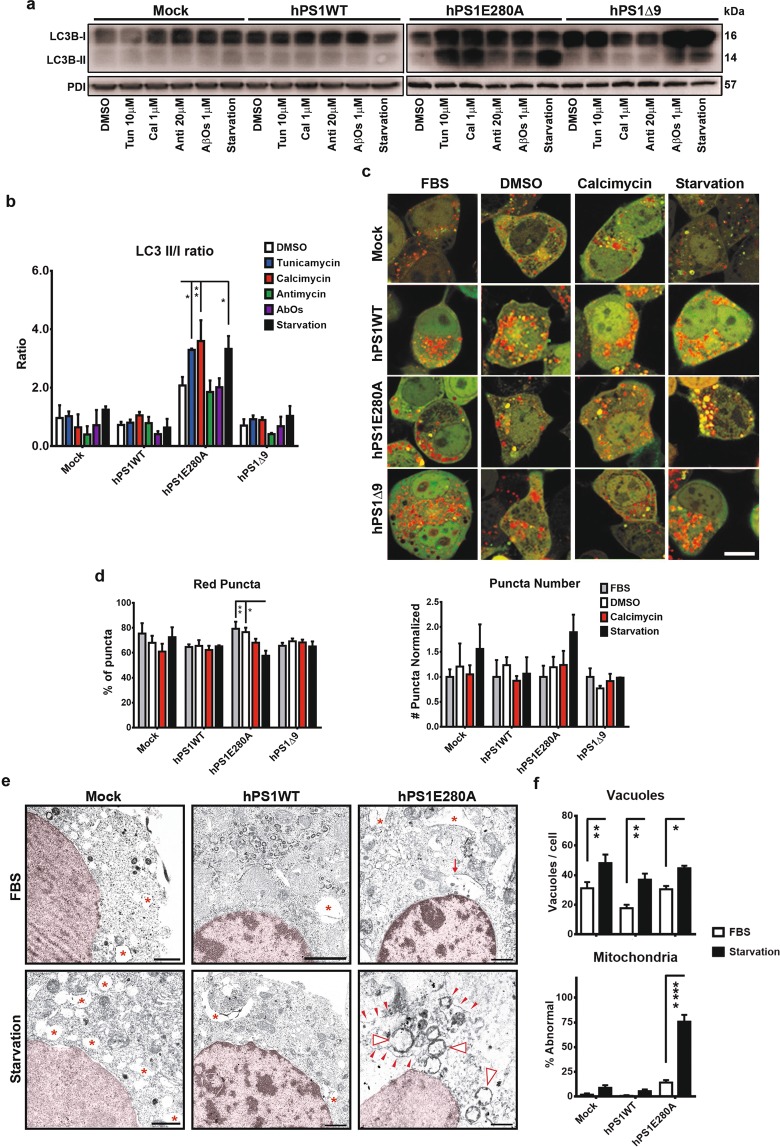
Abnormal autophagy in hPS1E280A N2a cells. (**a**) Steady state levels of LC3B I and II in N2a cells overexpressing hPS1, hPS1-E280A or hPS1Δ9 after tunicamycin, calcimycin, antimycin, AβOs, or serum starvation treatment. (**b**) LC3B densitometric quantification normalized to PDI and compared to basal conditions. LC3B conjugation (assessed by measuring LC3B-II/LC3B-I ratio) was increased in hPS1E280 cells after tunicamycin, calcimycin, and serum starvation treatments, while PS1Δ9 mutation showed no statistically significant differences. Mean ± SEM Two-Way ANOVA *p < 0.05, **p < 0.01, ***p < 0.001, ****p < 0.0001, n = 3. (**c**) Monitoring of autophagy turnover using N2a cells transfected with the RFP-GFP-LC3B construct. Transfected cells were treated with vehicle (DMSO) and stressors (calcimycin and serum starvation). The transition from autophagosomes (yellow) to autolysosomes (red) is detected by the loss of GFP fluorescence upon acidification of the autophagolysosome (for visualization of green and red channels, see Fig. S4). Bar = 1 μm. (**d**) Puncta quantifications showed significant differences in hPS1E280A cells by measuring whether red (decreased lysosomal degradation) or yellow (increased autophagosome formation) puncta. *P < 0.05, data are mean ± SEM, Two-Way ANOVA. (**e**) Ultrastructure analysis of basal and serum starved (16 h) mock, overexpressing hPS1WT, and hPS1E280A N2a cells (n = 3 independent experiments for each cell line). In basal conditions, hPS1E280A cells can show autophagic vacuoles but after 16 h of serum starvation, mitochondrial lysosomal degradation can be observed. Red asterisks = vacuoles, Red arrows = autophagic vacuoles, red arrowheads = lysosomal membrane, open arrowheads = degrading mitochondria. (**f**) Serum starvation significantly increased the number of vacuoles in all evaluated cell lines and only hPS1E280A cells it presented a significantly increased percentage of abnormal mitochondria (at least 10 cells and at least 130 mitochondria were evaluated from each cell line and condition). *P < 0.05, **P < 0.01, ****p < 0.0001; data are mean ± SEM, Two-Way ANOVA.

To assess the autophagic response in PS1 overexpressing N2a cells, we measured first steady state levels of LC3B-I and its conjugate, LC3B-II which serves as an indirect measurement of autophagic activity^27^. LC3B-II/I ratio was increased in hPS1E280 cells after tunicamycin, calcimycin and serum starvation treatments, whereas hPS1Δ9 cells showed no statistically significant differences (Figs. 2a,b and S4A). Increased levels of LC3B-II in hPS1E280A might reflect increased autophagic activity or impaired autophagic flux. Therefore, we evaluated the autophagic system in our cell lines in basal conditions, treated with positive stressors (calcimycin and serum starvation) or with calcimycin vehicle (DMSO) as its control; to finally transfect them transiently with a LC3B-GFP-RFP tandem construct. LC3B-RFP signal is more resistant to low pH, therefore the transition from the autophagosome to the autolysosome is reflected by the specific loss of GFP fluorescence upon acidification of the autophagolysosome (Figs. 2c and S4B). After 16 h of calcimycin and serum starvation, there were no significant changes in the autophagic turnover in mock, hPS1WT, and hPS1Δ9 cells. Only hPS1E280A cells showed significant differences between treatments with serum starvation by showing a lower proportion of red puncta when compared to basal conditions represented by serum presence or vehicle application (Figs. 2d and S5A). This difference was not reflected by the total number of puncta when normalized against puncta number in mock cells (Fig. 2d). It is possible that fewer red puncta indicates an alteration of autophagic flux, with the autophagosome not fusing successfully with the lysosome for degradation. To test this, we analyzed LC3B and LAMP1 co-localization as a marker of autophagolysosome fusion. There were no significant differences between un-starved or starved hPS1E280A N2a cells regarding LC3B-LAMP1 co-localization (Fig. S5B), suggesting that observed changes in red puncta numbers are due to pH modulation in those compartments rather than to autophagic flux deficiency. Finally, ultrastructural analysis of mock, hPS1WT and hPS1E280A overexpressing cells indicated that serum starvation lead to autophagic vacuoles formation in all cells but that only overexpression of hPS1E280A lead to increased lysosomal degradation of mitochondria under starvation and the presence of abnormal mitochondria (Fig. 2e,f). Previously, a role for PS1 in autolysosome acidification was reported, and defective autophagy was identified in PS1 mutants^28^. Our findings confirm this effect in hPS1E280A but not in hPS1Δ9 cells hinting towards a γ-secretase independent role regarding autophagy.

### Abnormal calcium homeostasis in hPS1 overexpressing N2a cells

**Figure 3:**
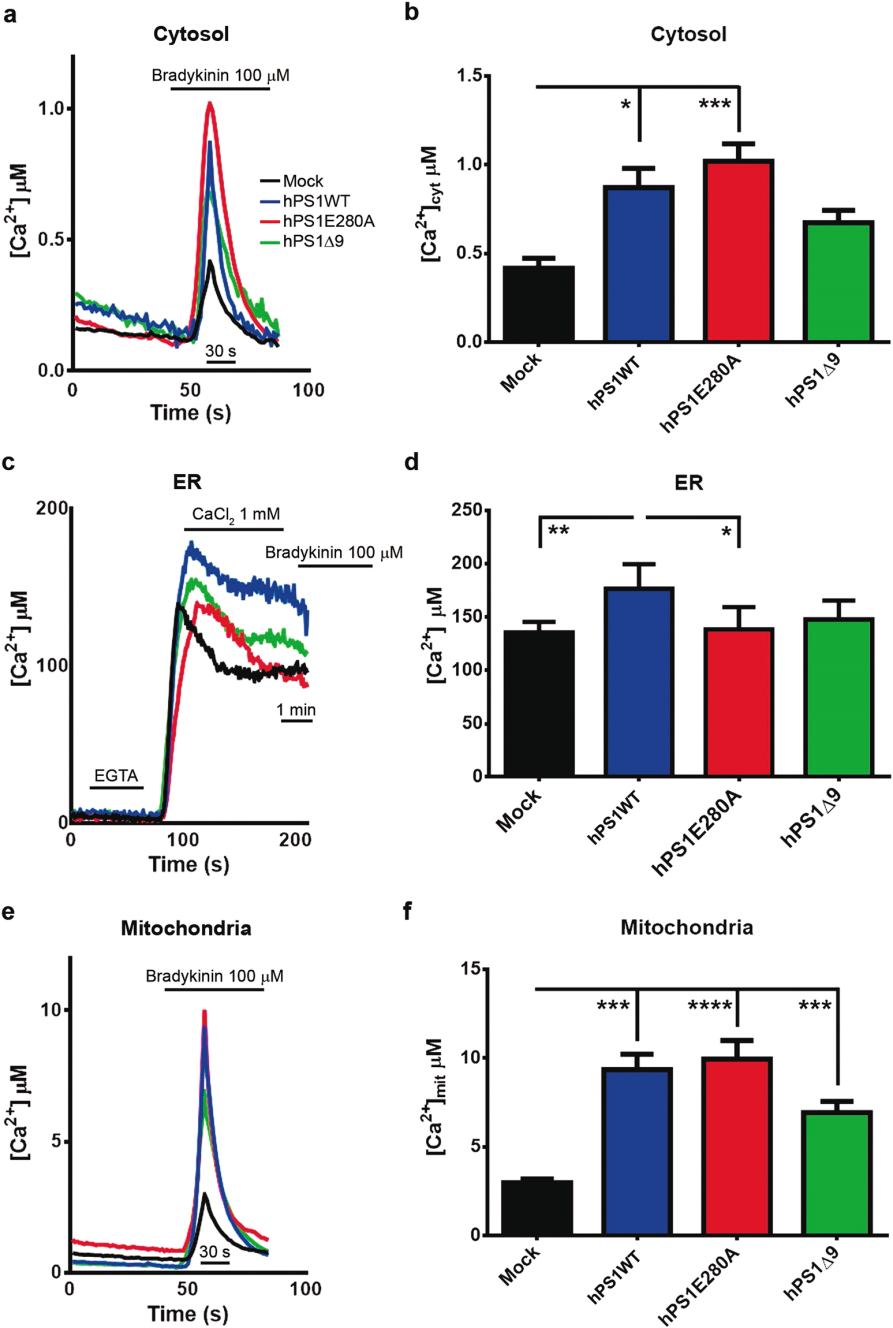
Abnormal calcium concentration in cellular compartments of N2a cells overexpressing wild type and mutanthPS1. N2a stably transfected cells overexpressing hPS1WT, hPS1E280A and hPS1Δ9 were used to measure intracellular calcium (Ca^2+^) concentrations in basal conditions using transient transfection of a compartment-specific aequorin constructs. Ca^2+^ intracellular levels were perturbed with the addition of 100 µM bradykinin for cytosolic and mitochondrial light recordings; for ER Ca^2+^ measurements, cells were first depleted of extracellular Ca^2+^ with EGTA, then intracellular Ca^2+^ concentrations were perturbed with the addition of 1 mM CaCl2. Finally, 100 µM bradykinin was added to deplete Ca^2+^ stores. (**a**) Representative averaged recordings of cytosolic calcium in the different cell lines, showing the maximum calcium concentration reached after Bradykinin addition. (**b**) Bar graphs of the maximum cytosolic calcium showing increased calcium concentration in hPS1WT and hPS1E280A cells. (**c**) Representative averaged recordings of ER calcium in N2a mock and hPS1 overexpressing cells. (**d**) Bar Graphs of maximal calcium concentration detected in the Endoplasmic Reticulum, hPS1WT showed increased calcium levels in the ER. (**e**) Representative averaged recordings of maximal mitochondrial calcium concentration in the different cell lines. (**f**) Bar graphs of the maximal mitochondrial calcium concentration after Bradykinin addition; mean and ±SEM are presented for all experiments, Two Way ANOVA, *p < 0.05, **p < 0.01, ***p < 0.001,****p < 0.0001 n = 3.

We have previously reported evidence of mitochondrial dysfunction and calcium dyshomeostasis in the cerebellum of FAD patients carrying PS1E280A mutation^23^. A cellular stress mechanism involving both processes could explain this observation. First, we used compartment specific calcium reporters to examine calcium homeostasis in our cellular model. In effect, cytosolic calcium concentrations were significantly increased in hPS1WT and hPS1E280A, when compared to mock N2a cells. hPS1Δ9, in contrast, showed decreased cytosolic calcium concentrations (Fig. 3a,b). ER calcium concentration was significantly higher only in hPS1WT N2a cells when compared to the others (Fig. 3c,d); and mitochondrial calcium concentration was significantly increased in all hPS1 overexpressing cells when compared to mock cells (Fig. 3e,f). Only hPS1WT cells presented elevated calcium levels in all compartments when compared to controls. Although increased ER calcium in hPS1WT could be attributed to PS1 overexpression, cells overexpressing mutant hPS1 did not present that effect in the ER. In fact, cytoplasmic and mitochondrial calcium levels showed a similar trend in which hPS1E280A calcium levels were significantly increased in both compartments when compared to controls. Finally, the overexpression of hPS1 seems to have a direct effect in mitochondrial calcium levels. The divergence between ER and mitochondrial calcium levels in hPS1E280A cells can potentially be associated to a mitochondria-specific alteration in this mutation. Interestingly, when PS1 is knock down in N2a cells, only mitochondrial calcium is affected with PS1KD cells showing significantly lower levels (Fig. S6A), indicating a direct role for PS1 in mitochondrial calcium homeostasis.

### Basal mitochondrial dysfunction in PS1 Mutant N2a cells and cellular stress

**Figure 4:**
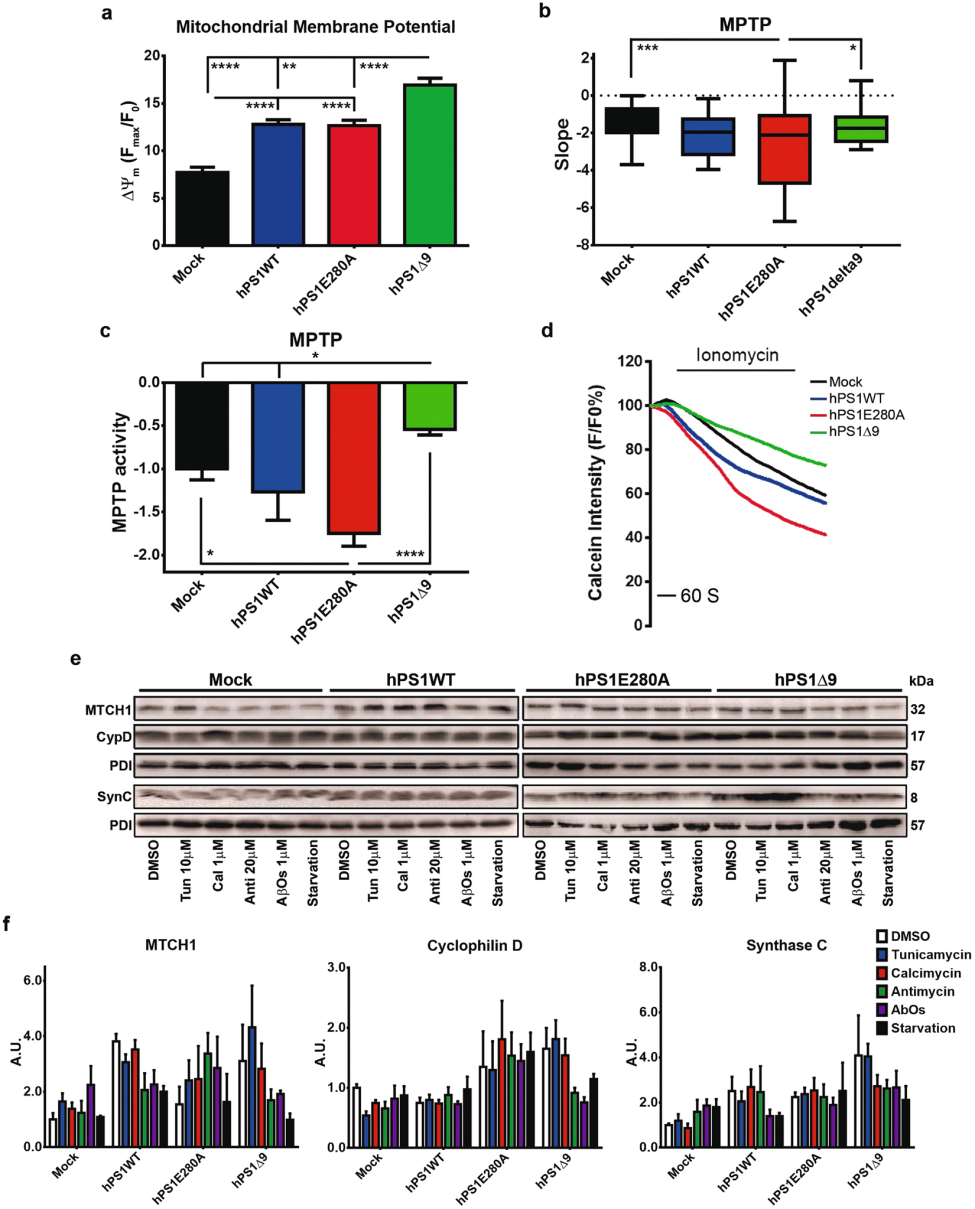
PS1 mutations lead to mitochondrial stress and abnormal mitochondrial permeability transition pore opening. (**a**) Bar graphs representing TMRM intensity as measurement of mitochondrial membrane potential (ΔΨ_m_) during live imaging. All hPS1 overexpressing cells showed increased potential compared to mock and hPS1Δ9 showed increased potential when compared to all other cell lines. (**b**) Cells were challenged with H_2_O_2_ 500 μM to induce mPTP opening and depolarize mitochondria, using TMRM intensity for assessment. hPS1E280A MPTP opening was accelerated compared to mock. SEM, **p < 0.01, ***p < 0.001, n = 93–158. (**c**) MPTP opening in N2a cells assessed with the Co^2+^ -calcein assay in three independent cell cultures of N2a cells overexpressing hPS1, hPS1-E280A or hPS1Δ9. Cells were challenged with 1 μM ionomycin (Sigma-Aldrich, Hamburg, Germany) to induce MPTP opening and quenching of the calcein signal. PS1 mutant cells showed altered MPTP opening, accelerated in hPS1E280A and inhibited in hPS1Δ9 cells. (**d**) Representative timeline of calcein intensity quenching after the addition of ionomycin in hPS1 overexpressing and mock N2a cells. (**e**) Representative western blots for MPTP (Cyclophilin D, CypD and ATP synthase C, SynC) or PS1 associated (MTCH1) mitochondrial proteins in N2a cells after stress treatments. (**f**) Bar graphs of densitometric analysis of steady state levels of CypD, SynC and MTCH1. Only hPS1WT overexpressing cells presented significantly increased MTCH1 levels. PS1-Mutants showed increased general steady state levels of CypD and SynC independent of stress. All data are mean ± SEM, Two-Way ANOVA, *p < 0.05, **p < 0.01, ***p < 0.001, ****p < 0.0001.

Alongside their multiple metabolic functions, mitochondria play a role in calcium homeostasis in the cell^20^. Following mitochondrial calcium measurements, we evaluated basal mitochondrial membrane potential (ΔΨ_m_) in mock and stably transfected hPS1N2a cells as a measurement of mitochondrial health. hPS1 overexpressing cells presented with significantly increased ΔΨ_m_ when compared to mock and hPS1Δ9 cells showed significantly higher ΔΨ_m_ when compared to hPS1WT and hPS1E280A cells (Fig. 4a). Furthermore, PS1KD N2a cells showed significantly decreased ΔΨ_m_ when compared to control cells (Fig. S6B). This, together with altered mitochondrial calcium concentrations, suggest a possible activation of mitochondrial permeability transition pore (MPTP). We evaluated MPTP opening in mock and PS1 overexpressing N2a cells, using two different methods. Assessment of MPTP-dependent alteration of the mitochondrial transmembrane potential showed that only hPS1E280A cells accelerated MPTP opening (Fig. 4b). By using the calcein-quenching assay, we found that only PS1 mutants, hPS1E280A and hPS1Δ9 cells, presented with significantly accelerated and delayed MPTP openings, respectively, when compared to mock cells (Fig. 4c,d). Both results indicate a PS1 mutation-dependent effect on MPTP activity in N2a cells. Furthermore, MPTP assessment using both methods showed no significant differences between PS1KD and control cells (Fig. S6B). Finally, we also applied our stress model and measured steady state levels of cyclophilin D and ATP synthase C, as putative MPTP components^29^, together with MTCH1, a mitochondrial PS1 interacting protein involved in apoptosis signaling^30^. Although no treatment-specific differences were identified (Fig. 4e,f), cyclophilin D showed significantly increased levels in hPS1 mutated cell lines when compared to mock and hPS1WT cells. Meanwhile, MTCH1 levels were significantly increased in hPS1WT but not in PS1 mutants and ATP synthase C was significantly increased in hPS1Δ9 cells (Supplementary Table 2). In summary, mitochondrial health findings support a role for PS1 in mitochondrial homeostasis with PS1 mutations potentially leading to mitochondrial stress characterized by abnormal MPTP opening in basal conditions.

### γ-secretase dependent and independent cellular stress phenotypes in PS1E280A overexpressing cells

**Figure 5:**
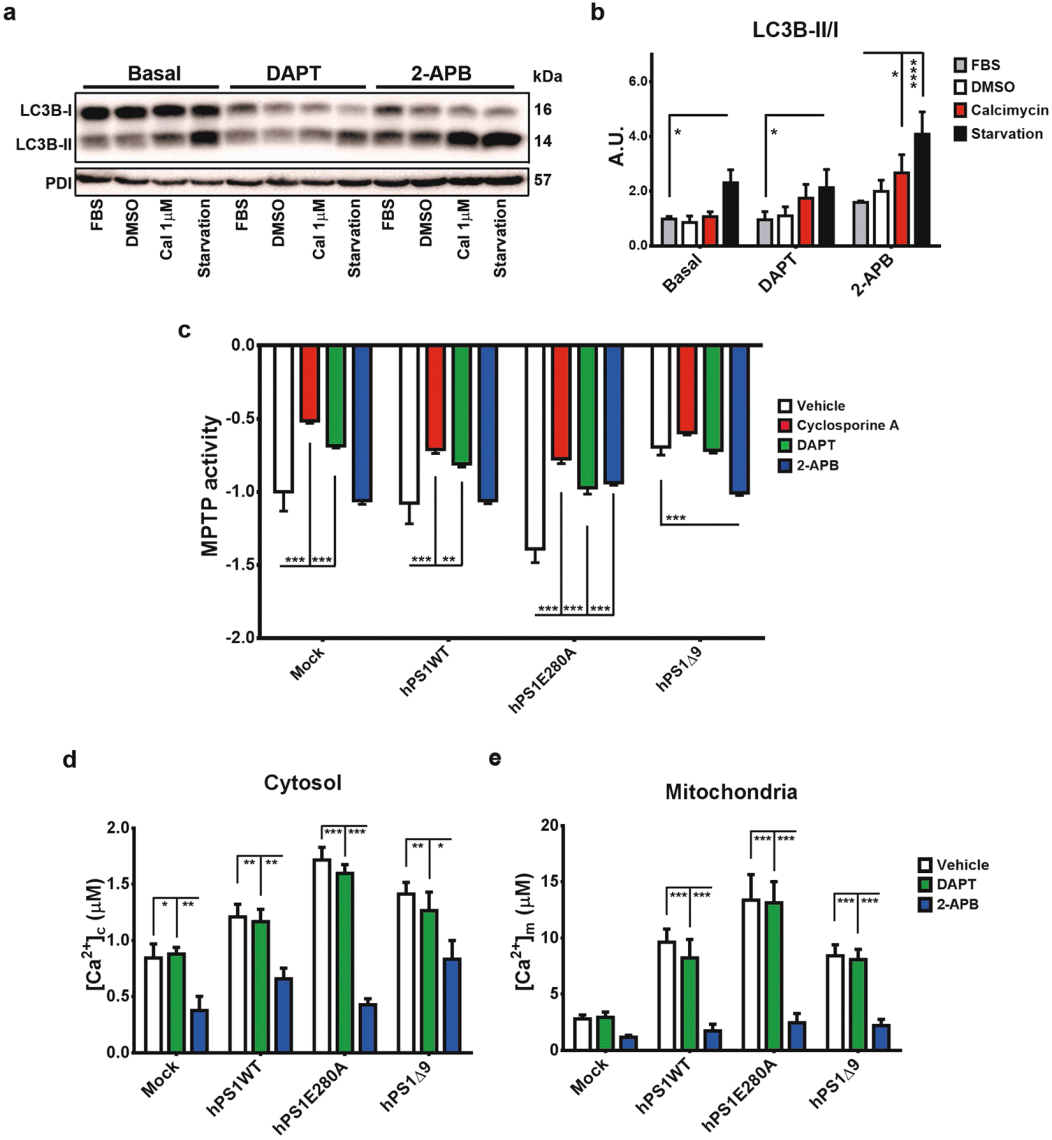
γ- secretase dependent and independent cellular stress response in hPS1E280A cells. (**a**) LC3B conjugation was evaluated in hPS1E280A cells treated with γ-secretase inhibitor DAPT and ER calcium channels inhibitor 2-APB. Representative western blot for LC3B from hPS1E280A N2a cells treated with FBS, DMSO, calcimycin and serum starved, all for 16 h. Cells were assessed with and without concurrent 16 h exposure to DAPT or 2-APB. (**b**) Bar graphs for densitometric analysis of hPS1E280A N2a cells. DAPT increased LC3B conjugation when compared to basal conditions and 2ABP increased it after calcymicin treatment or starvation in hPS1E280A cells. (**c**) Mock transfected, hPS1WT, hPS1E280A, and hPS1Δ9 N2a cells were challenged with 1 μM ionomycin to induce MPTP opening and quenching of the calcein signal. Cells were treated with Cyclosporin A, DAPT, or 2-APB. Cyclosporin A and DAPT inhibited MPTP opening in mock, hPS1WT, and hPS1E280A cells while 2-APB only showed an effect in PS1 mutants, inhibiting MPTP opening in hPS1E280A cells and accelerating it in hPS1Δ9 cells. (**d**) Bar graphs of maximum cytosolic calcium concentration and (**e**) mitochondrial calcium concentration in the different N2a cell lines, treated with DMSO (vehicle), DAPT and 2-APB for 16 h. 2-APB decreased mitochondrial calcium levels in PS1 overexpressing cells and cytoplasmic calcium levels in all cells. *P < 0.05, **P < 0.01, ***P < 0.001. Data are mean ± SEM, Two-Way ANOVA.

In our present study, we have found PS1E280A specific changes in autophagy, calcium homeostasis and mitochondrial health. To define if those changes are related to γ-secretase dysfunction due to this mutation and to differentiate between γ-secretase dependent and independent processes, we evaluated LC3B conversion and MPTP opening using N-[N-(3,5-Difluorophenacetyl)-L-alanyl]-S-phenylglycine t-butyl ester (DAPT), a γ-secretase inhibitor (Fig. S7A) and 2-Aminoethoxydiphenylborane (2-APB), an ER calcium channels inhibitor. Regarding LC3B conversion, neither DAPT nor 2-APB reduced LC3B conjugation in PS1E280A cells overloaded with calcium or serum starved. On the contrary, LC3B conversion was significantly elevated in starvation conditions with DAPT and 2-APB treatments, and after combined treatment with calcimycin and 2-APB (Fig. 5a,b). Regarding MPTP modulation and similar to Cyclosporin A, DAPT was able to significantly inhibit MPTP opening in mock, hPS1WT and hPS1E280A cells but not in hPS1Δ9 cells. On the other hand, 2-APB only affected MPTP opening in PS1 mutants, inhibiting it in hPS1E280A cells and accelerating it in hPS1Δ9 cells (Fig. 5c). The effect of γ-secretase inhibition on MPTP opening was confirmed by using another γ-secretase inhibitor, Compound W, in mock, hPS1WT and hPS1E280A cells (Fig. S7B). Given the possible effect of mitochondrial calcium levels in MPTP opening, we evaluated mitochondrial and cytoplasmic calcium concentration under DAPT and 2-APB treatments in hPS1 overexpressing cells. As expected, only 2-APB had an impact in calcium levels in both, mitochondria and cytoplasm, although mitochondrial calcium levels were unaffected by 2-APB in mock cells (Fig. 5d,e). These results indicated that the autophagic phenotype in hPS1E280A cells relies neither on γ-secretase activity nor on the availability of cytoplasmic calcium. It can also be concluded that γ-secretase activity influences MPTP opening and that this role seems to be affected by PS1 mutations. Finally, ER retention of calcium affecting MPTP opening after 2-APB treatment was only observed in PS1 mutants. This last mechanism possibly occurred due to calcium regulation in the ER and mitochondria.

## Discussion

Mutations in PS1 confer a 100% penetrance for familial Alzheimer’s disease, characterized by earlier disease onset, more severe pathology and associated to increase of Aβ 1–42 levels in brain tissue^2–4^. Presenilins are the catalytic subunit of γ-secretase and their mutations affect APP processing, favoring the generation of longer Aβ peptides. Thus, the severe pathology found in FAD cases is often considered to be a consequence of Aβ aggregation and toxicity^1^. Nevertheless, collected evidence has suggested cellular roles for PS1 independent on Aβ generation that could also be affected by its mutations, modulating cellular responses to stress^31–33^. For instance, some previous reports showed contradictory evidence for a role of PS1 or PS1 mutations in unfolded protein response (UPR) and ER stress^34–36^ (Supplementary Table 1). Our results indicate that the downregulation or mutation of PS1 do not affect ER stress in two independent cellular models. Also, it is known that ER stress is susceptible to calcium modulation in the cell^37^. It should be noted that in our PS1 overexpressing model, abnormal calcium levels in diverse cellular compartments were not enough to induce ER stress or differential activation of it. Thus, previous evidence in favor or against a role of PS1 in ER stress should be further reassessed according to the cellular model used.

Autophagy is a physiological process for recycling diverse cellular constituents. However, under stress conditions, autophagy can be considered a pathogenic factor as seen in several neurodegenerative disorders. Disrupted autophagy leads to accumulation of autophagic vacuoles inside swollen dystrophic neurites in AD. Lysosome system failure during autophagy has been also associated to several AD pathological outcomes such as amyloidogenesis, neuritic dystrophy, apoptosis, and tauopathy^38^. Furthermore, autophagy dysfunction in AD is considered to be an effect of Aβ accumulation and toxicity^39^. We did confirm an effect of mutated PS1 in autophagy. PS1E280A showed a clear phenotype for autophagic dysfunction in N2a cells. Previous reports indicated that PS1 modulates autophagosome acidification regulating calcium homeostasis in the lysosome^12^. Interestingly, PS1Δ9 did not affect autophagy in our model. Deletion of exon 9 in PS1 has shown to affect γ-secretase dependent functions without affecting γ-secretase independent ones^40^. Lee *et al*. attributed PS1-mediated autophagic dysfunction to two γ-secretase independent processes: calcium homeostasis maintenance and lysosomal proteolysis^28^. We agree with this model, given that PS1Δ9 mutants do show impairment of γ-secretase dependent functions and that autophagy was unaffected in hPS1Δ9 N2a cells. Furthermore, increased LC3B conjugation was not rescued to normal levels by DAPT or 2-APB in hPS1E280A cells. Lack of effect of 2-APB also supports an independent regulatory function for PS1 in lysosomal calcium, independent on ER calcium retention. Previous studies^12,28^ and our results indicate that an underlying autophagic dysfunction can be an ongoing cellular process in PS1 mutation carriers, turning affected cells more vulnerable to other stressors, such as Aβ accumulation itself.

One of the mechanisms proposed for autophagy regulation via PS1 is the maintenance of lysosomal calcium homeostasis^28^. This mechanism is only one among several cellular calcium-related processes in which PS1 can play a role^7^. In fact, organelle calcium homeostasis seems to be a very relevant function for PS1, considering our findings on mitochondrial calcium levels and MPTP opening. Our measurements of total calcium and ER calcium for hPS1E280A cells went along with similar findings with other PS1 point mutations^41^ and confirmed the findings for PS1Δ9^42^. Regarding mitochondrial calcium concentration, PS1 overexpression in general showed an opposite effect to that reported for PS2 overexpression, which decreases mitochondrial calcium concentration in SHSY5Y cells^43^. It should be noted that increased mitochondrial calcium levels in hPS1 overexpressing cells could be associated to elevated ΔΨ_m_, and both phenomena could be associated to hPS1 overexpression in this cellular model. This association was further confirmed by decreased mitochondrial calcium and ΔΨ_m_ in PS1KD cells. However, PS1 mutant cells responded abnormally to calcium overload with PS1E280A accelerating and PS1Δ9 decelerating MPTP opening, while it was unaffected in PS1KD cells. Assembly and activation of MPTP is part of mitochondrial stress responses and can potentially lead to apoptosis signaling. However, transient MPTP activation seems to be a homeostatic mitochondrial response^44^. It has been suggested that Aβ toxicity can affect MPTP, whether indirectly via oxidative stress or directly, increasing Cyclophilin D translocation to the internal mitochondrial membrane and favoring MPTP opening^24^. Also, a mathematical model has been proposed linking abnormal calcium signaling, Aβ deposition and MPTP related apoptosis^45^. Remarkably, Aβ toxicity and pathology are absent in our model. Yet, cells overexpressing mutant hPS1 present with abnormal MPTP activity.

More to the point, γ-secretase inhibitors such as DAPT and Compound W effectively inhibit MPTP opening in mock, hPS1WT and hPS1E280A cells pointing to a role for γ-secretase in MPTP modulation. PS1 mutations effect on MPTP opening could be attributed to gain of abnormal function more than to loss of function, given that decreased PS1 levels did not modify MPTP opening in PS1KD cells, while γ-secretase inhibition successfully neutralized it. Decreased MPTP in hPS1Δ9 cells can be associated with significantly higher ΔΨ_m_. However, γ-secretase dysfunction in hPS1Δ9 cells could also explain MPTP inhibition. Additionally, 2-APB affected MPTP opening only in PS1 mutants, again with contrasting effects between the two PS1 mutations. In those cells, 2-APB effectively compensated abnormal basal MPTP profiles while decreasing mitochondrial and cytoplasmic calcium levels. Therefore, calcium retention in the ER relieves mitochondrial calcium overload in PS1 mutants which avoids abnormal MPTP opening. This effect can be achieved via mitochondria-ER interaction, given calcium exchange between these two organelles as previously suggested by Toglia *et al*. in their model^46^ and the known effect of 2-APB in mitochondrial calcium^47^. Mitochondrial function seems to be also altered in AD affecting lipid synthesis, respiratory chain, and calcium homeostasis^21^. Previous studies suggest increased ER-Mitochondrial apposition leading to augmented calcium trafficking between the two organelles as one possible explanation for mitochondrial dysfunction in AD^48^, that could lead to mitochondrial stress and cell death. This model of mitochondrial dysfunction in AD also portrays Aβ accumulation and toxicity as a causative factor^49,50^.

Independently of its wide use, hPS1 overexpression in otherwise unmodified murine cells can always bring unexpected effects given specific functional profiles of human vs murine Presenilins^51^. Our N2a hPS1 overexpression model has this limitation and our results should be interpreted within this context. Nevertheless, we observed specific γ-secretase dependent and independent dysfunction in overexpressing hPS1 mutants, with direct impact in some stress pathways. Also, although our experimental design aimed to induce cellular stress but not cellular death, findings regarding autophagy dysfunction in hPS1E280A cells and mitochondrial calcium-dependent stress in both hPS1 mutants suggested the possibility that cellular death pathways could be activated whether basally or via cellular stress induction. Remarkably, neither the treatment with antimycin nor Aβ 1–42 oligomers presented evidence for increased cellular stress in our model and assessed markers after 16 h. Doses used for both treatments are within range for stress induction in N2a cells, according to previous reports^52,53^, and oligomeric synthetic Aβ 1–42 is known to induce different types of cellular stress, sometimes simultaneously, in various cellular models^54,55^. It is possible that 16 h of treatment were not enough to allow these reagents to induce visible effects in our assays. As a confirmation of this, levels of the apoptotic marker pRIPK3 were significantly increased in mock N2a cells after 24 h of treatment with both, antimycin and Aβ 1–42 oligomers (Fig. S7C). Given that our purpose was to examine early stress events caused by PS1 mutations, we consider Aβ negative findings at 16 h to be a confirmation of a role for PS1 independent of Aβ toxicity in cellular stress. Likewise, lack of effect of antimycin and 16 h shows, at this point, that mutant PS1 effects in mitochondria are not directly related or modified by oxidative stress. Finally, additional limitations of our study include the use of a single cellular model, N2a cells, and of single stably transfected clones in our cell lines. These limitations should be taken in account for the interpretation of the results and for further exploration of our findings.

Previously, an elegant study by Guo *et al*. in a murine knock-in model determined that PS1 mutations confer increased neuronal vulnerability to excitotoxicity via apoptosis in hippocampal cells. They also showed that impaired mitochondrial function increased susceptibility to Aβ-induced stress^56^. The same group determined in PS1 transfected PC12 cells increased sensitivity to mitochondrial stress with associated elevated calcium in PS1 mutants. Interestingly, Cyclosporin A was used successfully to prevent apoptosis caused by oxidative stress in their model and already then, MPTP activation was suggested as a possible mechanism of increased mitochondrial stress in PS1 mutants^57^. More to the point, recently Toglia *et al*. suggested a theoretical model for the involvement of MPTP in PS1 mutants as a result of IP3R altered activity and increased mitochondrial calcium uptake^46^. We have confirmed both, previous findings and the theoretical model, but we bring in the novel finding of γ-secretase as a direct modulator of MPTP independent of mitochondrial calcium homeostasis.

In this study we presented an alternative mechanism for mitochondrial damage in PS1 FAD with altered MPTP as a driving force behind it, and we showed how PS1 mutations affect cellular stress responses simultaneously with specific mutations presenting different profiles. Although several of these stress mechanisms have been reported previously to be altered in different cellular models, our experimental design allowed us to integrate them on a single picture with PS1 mutations simultaneously affecting calcium homeostasis, lysosomal function and mitochondria. As with autophagy, PS1 mutation-dependent alterations in mitochondria detected in our model occur under basal conditions or as a result of acute non-lethal cellular stress. We have previously reported evidence of mitochondrial dysfunction in PS1E280A patients, reproduced on animal and cellular models^23^. Our results confirm that PS1 mutations render the cell more susceptible to different kinds of cellular stress, such as autophagy or mitochondrial dysfunction, on a mutation-specific manner. Even in one single mutation, stress pathways involving γ-secretase dependent and independent mechanisms simultaneously can be identified without the participation of Aβ toxicity. These Aβ-independent stress mechanisms point to a larger role of PS1 on cellular homeostasis and death beyond APP processing. We suggest that in PS1 FAD patients basal cellular stress could be taking place throughout life, increasing vulnerability to damage in susceptible cells and presenting eventually with cumulative effects leading to the complex end-point pathology found in AD. The identification of cellular stress mechanisms in human post mortem brain tissue after a long degenerative process is complicated further by multiple events that lead to the end-point pathology found in the tissue. Therefore, we consider that the study of such mechanism in cellular models is still a valid approach to reconstruct the pathophysiology observed in FAD. Although further studies in cellular and animal models need to be done, we suggest the possibility that earlier disease onset, more severe brain atrophy and underlying neurodegeneration in FAD are a result not only of earlier Aβ pathology but of latent cellular vulnerability to stress due to other PS1 functions.

## Materials and Methods

### Cellular culture and transfection

Murine Neuroblastoma N2a cells were stably transfected with pcDNA 3.1 Zeo + vector with resistance to Zeocin. 1 µg of 4 different plasmids: Mock (empty vector), human PS1WT, PS1E280A and PS1Δ9 were used to transfect the cells with lipofectamine^TM^ 2000 (Thermo Fisher Scientific, Schwerte, Germany), according to manufacturer’s instructions. The clones were established by selection with Zeocin (Invitrogen, Carlsbad, CA, USA). The dose used for selection was 200 µg/ml. Positive clones were isolated after approximately 30 days. Overexpression of human PS1 was assessed via western blot and qPCR for human PS1 (Fig. S1A–C). Cells were cultured in Dulbecco’s Modified Eagle Medium (DMEM) supplemented with 10% fetal bovine serum (FBS) and treated with different reagents to induce cellular stress and death (see below). *PS KO*−/− MEFs and *PS KO*−/− MEFs stably transfected with wildtype or mutated (E280A) human PS1 were kindly donated by Bart de Strooper (VIB, Leuven, Belgium). This cellular model has been used previously to study γ-secretase function^58^ and to study Presenilins impact in mitochondrial function^59^. MEFs were cultured in DMEM supplemented with 10% FBS at 37 °C and 5% CO_2_ and treated with tunicamycin and calcimycin 10 µM.

### Reagents

Cell stress inducers were dissolved first in DMSO (maximal concentration 0.1%) and added to cells as follows: ER stress with 10 µM tunicamycin (Sigma-Aldrich, Hamburg, Germany), calcium overload with 1 μM calcimycin (Sigma-Aldrich, Hamburg, Germany). Oxidative stress with 20 µM antimycin (Sigma-Aldrich, Hamburg, Germany), autophagy induction via serum starvation (DMEM without FBS), Aβ toxicity with 1 µM Aβ 1–42 oligomers (see oligomers preparation). Also, 250 nM of N-[N-(3,5-Difluorophenacetyl)-L-alanyl]-S-phenylglycine t-butyl ester (DAPT, Sigma-Aldrich, Hamburg, Germany) and 10μM of Compound W (Tocris Biosciences, Bristol, England), γ-secretase inhibitors, were used to evaluate the role of γ-secretase activity in cellular stress and MPTP opening. Furthermore, 100 μM of 2-Aminoethoxydiphenyl borate (2-APB, Sigma-Aldrich, Hamburg, Germany), an ER calcium channels inhibitor, was used to evaluate the influence of store operated calcium and 1.6 μM Cyclosporin A, was used as a MPTP inhibitor (Sigma-Aldrich, Hamburg, Germany) (see Supplementary Table 3 for mechanisms of action).

### Aβ oligomers preparation

Aβ 1–42 synthetic peptide oligomerisation was conducted according to published protocols^60^. Lyophilized powder of Synthetic human Aβ 1–42 (GenicBio, Shanghai, China) was first dissolved to 1 mM solution by adding 1,1,1,3,3,3-Hexafluoro-2-Propanol (HFIP). Aβ-HFIP solution was incubated at room temperature (RT) for at least 30 min and then the tubes were opened to allow HFIP evaporate overnight. Tubes were transferred to a SpeedVac and dried down without heating for 1 h to remove any remaining traces of HFIP. A thin clear film at the bottom of the tubes was obtained and diluted again whether by adding DMSO (final concentration 5 mM) to store at −20 °C or DMSO (5 mM) plus Medium (DMEM + FBS 10%) for a final concentration 1 µM for cell treatment.

### Western blots

Cells were harvested after 16 h of treatment for each reagent and lysed with a buffer containing 50 mM Tris-HCl, 150 mM NaCl, 1 mM Ethylenediaminetetraacetic acid (EDTA), 10% glycerol,1% NP-40 and sodium azide. Protein concentration was determined with the bicinchoninic acid method, and equal amounts of protein were loaded into a 12% sodium dodecyl sulfate–polyacrylamide gel electrophoresis and transferred to a Polyvinylidene difluoride membrane. Membranes were blocked with 5% non-fat milk + 0.1% Tween 20 in tris-buffered saline for 1 hour and then incubated with specific primary antibodies overnight at 4 °C. Early and late markers for ER stress, mitochondrial proteins, and autophagy were assessed by immunoblotting (see Supplementary Table 4). Afterwards blots were incubated with secondary antibodies for 1 hour and detected by chemiluminescence. Densitometric analysis was performed using Image Studio Lite 5.2 (LI-COR, Lincoln, NE, USA).

### Autophagic turnover assessment

All cell types were transiently transfected using Lipofectamine^TM^ 2000 with a tandem LC3B fluorescent construct including Green fluorescent protein (GFP) and Red fluorescent protein (RFP) (pMRX-IP-GFP-LC3-RFP Plasmid, Addgene, Cambridge, MA, USA) following the manufacturer´s instructions. Autophagic turnover assessment was performed after 16 hours of incubation with calcimycin or serum starvation, according to methods described above, cells treated with DMSO or only culture medium were used as controls. The cells were visualized using a Leica LCS-SP5 confocal microscope system, all experiments were performed under live imaging conditions (SP5 confocal microscopy chamber at 37 °C and 5% CO_2_). Transfected LC3B could be detected as emitting in the green and red channels (yellow signal) if it was located in the cytoplasm or autophagosomes; and as emitting in the red channel only in autolysosomes^27^.

### Ultrastructural analysis

Mock, hPS1WT and hPS1E280A N2a cells were collected and centrifuged in PBS at 1000 g for 5 minutes at 4 °C. The resulting pellets were prefixed with 0.25% glutaraldehyde and 4% paraformaldehyde in PBS, for 24 h at 4 °C. Prefixed pellets were further fixed in glutaraldehyde and chrome-osmium at least for 2 hours at room temperature, dehydrated in ethanol, and embedded in Epon 812 (Serva Electrophoresis GmbH). After polymerization, 1-μm-thick sections were cut, stained with toluidine blue, and checked for adequate cell morphology. For further processing for electron microscopy, relevant specimens were cut into 60- to 80-nm-thick sections, which were contrasted with uranyl acetate and lead solution. Sections were visualized with a LEO EM 912AB electron microscope (Carl Zeiss GmbH, Jena, Germany).

### Intracellular calcium measurements

Targeted aequorins (AEQs) were used to measure intracellular calcium concentrations in N2a cells. 50000 cells were grown in coverslips and transfected with 0.5 µg aequorin-cDNA construct directed to different cellular compartments (cytAEQ, mitAEQ and ERAEQ, described in^61^). Mitochondrial and cytosolic responses were induced by the addition of 100 μM Bradykinin (Sigma-Aldrich, Hamburg, Germany). ER calcium re-uptake was measured in calcium depleted cells. Cells were lysed with a 10 mM calcium and 0.1%Triton containing buffer at the end of the experiment to estimate the efficiency of transfection as described previously^61^.

### Mitochondrial membrane potential measurement

Mitochondrial membrane potential (ΔΨ_m_) was measured by loading cells with 10 nM tetramethyl rhodamine Methylester (TMRM, Thermo Fisher Scientific, Schwerte, Germany) for 30 min at 37 °C. Images were taken with an inverted microscope (NikonLiveScan Swept Field Confocal Microscope (SFC) Eclipse Ti equipped with NIS-Elements microscope imaging software, Nikon Instruments, Amstelveen, Netherlands). TMRM excitation was performed at 560 nm and emission was collected through a 590 to 650 nm band-pass filter. Images were taken every 5 s with a fixed 20 ms exposure time. 10 µM carbonyl cyanide p-trifluoromethoxyphenylhydrazone (FCCP, Sigma-Aldrich, Hamburg, Germany), an uncoupler of oxidative phosphorylation, was added after 5 minutes of acquisitions in order to completely collapse the electrical gradient established by the respiratory chain. ΔΨ_m_ was determined as the ratio of basal TMRM fluorescent signal intensity (F_max_) divided by TMRM fluorescent signal intensity after FCCP addition (F_0_).

### Assessment of mitochondrial transition pore opening

#### MPTP assessment by Calcein/Co^2+^quenching assay

Permeability transition pore complex opening was assayed as previously described^62^. Cells were loaded with 1 mM calcein acetoxymethyl ester (Sigma-Aldrich, Hamburg, Germany) and Co^2+^ as instructed by the Image-IT® LIVE Mitochondrial Transition Pore Assay Kit (Thermo Fischer Scientific, Schwerte, Germany). Cells were then imaged based on 490 ± 20 nm excitation and 525 nm long pass emission filters with an Axiovert 200 M fluorescence microscope equipped with a 40X water immersion objective (N.A. 1.2, from Carl Zeiss Microscopy, Jena, Germany). Finally, images were analyzed with MetaMorph® (Molecular Devices, LLC, San Jose, CA, USA), and quenching rate was calculated as the slope of the fluorescence trace over a period of 60 sec after stimulation.

#### MPTP assessment by Mitochondrial transmembrane potential

Assessment of MPTP opening via ΔΨ_m_ was performed as previously described^63^. Cells were loaded with 10 nM TMRM (Thermo Fischer Scientific, Schwerte, Germany) in Krebs-Ringer buffer supplemented with 250 μM sulfinpyrazone, then placed in a humidified chamber at 37 °C and imaged with a LiveScan Swept Field Confocal Microscope (Nikon Instruments, Inc.) equipped with a 60× oil immersion (N.A. 1.4, from Nikon Instruments, Inc.) every 30 sec for 30 min. TMRM fluorescence was analysed with the NIS Elements software package (Nikon Instruments, Amstelveen, Netherlands), and depolarization rate were calculated as the slope of the fluorescence trace over a period of 10 min after stimulation.

### Statistics

Data were analyzed by GraphPad Software (La Jolla, CA, USA) using one-way or two-way ANOVA, when required, followed by Holms-Sidak post-hoc correction, values are given as mean ± standard error of mean (SEM). Differences with p-value < 0.05 were considered significant.

## Supplementary information

Supplementary Methods and Results.

## Data Availability

Relevant data for the interpretation of the results is included in the manuscript and supplementary files. More detailed data and specific materials such as cell lines used, are available from the corresponding author on reasonable request.
