# Supplementary material for: Susceptibility to cellular stress in PS1 mutant N2a cells is associated with mitochondrial defects and altered calcium homeostasis
Source: Sci Rep. 2020 Apr 15;10:6455. doi: 10.1038/s41598-020-63254-7 (PMC7160112; doi:10.1038/s41598-020-63254-7)
Supplement: Supplementary file 1 — Supplementary Methods and Results. [file 41598_2020_63254_MOESM1_ESM.pdf]

Susceptibility to cellular stress in PS1 mutant N2a cells is associated with mitochondrial defects and altered calcium homeostasis

Liliana Rojas-Charry*[1], Sergio Calero-Martinez*[1], Claudia Morganti[2], Giampaolo Morciano[2], Kyungeun Park[1], Christian Hagel[1], Stefan J. Marciniak[3], Markus Glatzel[1], Paolo Pinton[2] and Diego Sepulveda-Falla[1#]

1. Institute of Neuropathology, University Medical Center Hamburg-Eppendorf, Hamburg, Germany. 2. Department of Morphology, Surgery and Experimental Medicine, Section of Pathology, Oncology and Experimental Biology, University of Ferrara, 44121, Ferrara, Italy. 3. Cambridge Institute for Medical Research (CIMR), University of Cambridge, Cambridge, UK. * These authors contributed equally. # Correspondence to be addressed to: Dr. Diego Sepulveda-Falla, 05.002, Institute of Neuropathology, N27, University Medical Center Hamburg-Eppendorf, Martinistrasse 52, 20246. Hamburg, Germany. E-mail: dsepulve@uke.de

Supplemental Material and Methods

Quantitative PCR (qPCR)

Total RNA was isolated from cells with RNeasy Mini Kit (Qiagen, Hilden, Germany) according to the manufacturer's instructions (Invitrogen, Carlsbad, CA, USA). The isolated RNA was then treated with DNase I (Invitrogen, Carlsbad, CA, USA). 500 ng of total RNA was reverse transcribed with RevertAid H Minus First Strand cDNA Synthesis kit (Thermo Fisher Scientific, Schwerte, Germany) using OligodT´s. cDNA templates were diluted ten-fold before performing qPCR. The primers used for PS1 were 5'-CTGGTGAAGACCCAGAGGAA-3' (forward) and 5'-AAACAAGCCCAAAGGTGATG-3' (reverse) while the primers for β-actin were 5´-GCTTCGCTGTCTACTTTCCA-3' (forward) and 5´- CAGCCCGACTACTGCTTAGA-3´ (reverse) (Greenough et al., 2011). Quantitative PCR was performed on a RotorGeneQ (Qiagen, Hilden, Germany) with Maxima SYBR Green/Fluorescein qPCR Master Mix (Thermo Fisher Scientific, Schwerte, Germany). Results were analyzed using the Delta-Delta-Ct method (Kubista et al., 2006; Livak and Schmittgen, 2001).

Confocal Immunofluorescence

For confocal microscopy cells were cultured and fixed with pre-warmed 4% paraformaldehyde for 10 min, then washed three times with Phosphate buffered saline (PBS) 1X for 5 min and permeabilized with Triton X-100 0.25% in PBS 1X for 10 min. Blocking buffer (1% BSA and Triton X-100 0.25% in PBS 1X) was added for 1 h. Then the cells were incubated overnight with antibodies against PS1, KDEL, Tom20, GM130, LC3B and LAMP1. The primary antibody was washed with PBS 1X 3 times for 5 min. The secondary fluorescent antibody (Alexa Fluor 555 goat anti-rabbit IgG, 1:500, A-11079 Invitrogen, Carlsbad, CA, USA and Alexa Fluor 488) was added for 2 h. Then the cells were washed again and one drop of Fluoromount-G™ was applied to each slide. Images were acquired on a LCS-SP5 (Leica

Microsystems, Wetzlar, Germany) confocal microscope, with a HCX PL APO Lambda blue 63x/1.4 Oil UV objective under illumination with a HeNe laser at 488 nm. Pictures were obtained by using z-stacks of 20 images separated by 1 µm along the z axis. Colocalization was analyzed using Image J software 1.51d (National Institutes of Health, Baltimore, MD, USA). For LC3B and LAMP1 colocalization, serum starved (16 h) and non-starved PS1E280A N2a cells were cultured, fixed and analyzed as described above.

Murine Aβ 1-40 and Aβ 1-42 ELISA assay

Sandwich enzyme-linked immunosorbent assay (ELISA) for murine Aβ 1-40 and Aβ 1-42 was performed as recommended by the manufacturer (kits KMB3481 and KMB3441, Thermo Fisher Scientific, Schwerte, Germany). N2a cells overexpressing hPS1 and mock transfected cells were harvested and lysed with lysis buffer (50mM Tris-HCl, 150mM NaCl, 1mM EDTA, 10% glycerol,1% NP-40 and sodium azide). Protein concentration was determined with the BCA method, and equal amounts of protein were used for Aβ peptides concentration assessment. Protein lysates were collected and probed with the ELISA kit for each antigen. Samples were measured at 450 nm in a Bio-tek mQuant spectrophotometer (Winooski, VT, USA) and expressed as ng/mg of total protein.

Murine PS1 knock down in N2a cells

Murine Neuroblastoma N2a cells were transfected using Lipofectaime RNAiMAX reagent (Thermo Fisher Scientific, Schwerte, Germany) following the manufacturer's instructions. To Knock down PS1, cells were transfected with 20uM PS1 siRNA MSS208051 together with RNAiMAX diluted in OPTIMEM medium and added to DMEM + FBS medium for 48 hours. For negative control, cells were similarly transfected using 8nM Stealth siRNA negative control. Both, PS1 and negative control siRNA were acquired from Thermo Fisher Scientific, Schwerte, Germany. Transfection efficiency of PS1 siRNA was evaluated by western blot after each experiment. Out of 36 transfections we observe mean PS1 levels = 13.52 %, max = 27.84 %, min = 0.51 %, standard deviation = 7.16 % (Fig. S3A-B).

1. Greenough, M.A. *et al.* Presenilins promote the cellular uptake of copper and zinc and maintain copper chaperone of SOD1-dependent copper/zinc superoxide dismutase activity. *J. Biol. Chem.* **286**, 9776-86 (2011).

Table 1

Author	Journal	Year	Model	Result
**Niwa**	Cell	1999	COS1/Hela cells	Impaired translocation of IRE1 by PS1 Kn in activated UPR
Katayama	NatCell Biol.	1999	SK-N-SH neuroblastoma cells	PS1 mutationsdecreaseBiP
**Sato**	NatCell Biol.	2000	Primary cultured fibroblasts from Ps1 mutant mice /N2a cells	No effect
Imaizumi	NatCell Biol.	2001	SK-N-SH neuroblastoma cells	Inhibited IRE1 and PERK in PS1 mutants
Imaizumi	BiochemBiophys Acta	2001	-----	Inhibited IRE1 and BiP in PS1 mutants (review)
**Katayama**	JBC	2001	SK-N-SH neuroblastoma/MEFs cells	PS1 mutants inhibit Ire and ATF6
Sato	JBC	2001	SK-N-SH neuroblastoma/N2a cells	impaired UPR (Bip)
**Siman**	JBC	2001	Primary cultured cortical neurons	No effect after 16h of treatment
Steiner	NeurobiolDis	2001	HEK293 cells	ATF6 not affected by PS1
Chan	NeurobiolDis	2002	PC12 cells / Primary cultured hippocampal neurons	PS1 mutants increased caspase 12 and calpain levels
Milhavet	J Neurochem	2002	PC12 cells/ Knock-in mice	Increased CHOP and eif2α
Terro	J Neurosci Res	2002	Primary cultured cortical neurons	PS1 mutants are more sensitive to ER stress death
Yasuda	BBRC	2002	N2a cells/primary cultured fibroblasts from mutant knock-in PS1 mice	PS1 mutantsinhibit PERK
Katayama	J ChemNeuroanat	2004	-----	Review of previous
Piccini	NeurobiolDis	2004	Primary cultured fibroblasts from FAD patients	No effect
Hoozeman	Acta Neuropathol	2005	Post-mortem brain tissue	Increased BiP and p-Perk
Yukioka	NeurochemInt	2008	SK-N-SH Neuroblastoma/COS7 cells	IncreasedCaspase 4 activation
Jin	JAD	2010	HEK293/GOTO/KNS-42 cells	Tunicamycin increases PS1 andBiP levels. PS1 protects through interactionwith SERCA
Szaraz P	ArchBiochemBiophys	2013	HepG2	PS1 silencing increased BiP, PDI, CHOP, ATF6. Also lead to autophagosome formation.
Shao Y	Int J ClinExp Med	2015	SH-SY5Y	PS1 mutant decreased BiP levels

Color code: Blue shade = decreased response, Pink shade = increased response, Green shade = no changes.

Table 2

Protein	Line comparison	p Value
GADD34	hPS1WT vs. Mock	0.6269
	hPS1E280A vs. Mock	0.5385
	hPS1D9 vs. Mock	0.6336
	hPS1E280A vs. hPS1WT	0.9812
	hPS1D9 vs. hPS1WT	0. 9812
	hPS1D9 vs. hPS1E280A	0. 9812
BiP	hPS1WT vs. Mock	0.9080
	hPS1E280A vs. Mock	0. 9080
	hPS1D9 vs. Mock	0.8555
	hPS1E280A vs. hPS1WT	0.9829
	hPS1D9 vs. hPS1WT	0. 9829
	hPS1D9 vs. hPS1E280A	0. 9829
CHOP	hPS1WT vs. Mock	0.5130
	hPS1E280A vs. Mock	0.4236
	hPS1D9 vs. Mock	0.7982
	hPS1E280A vs. hPS1WT	0.8354
	hPS1D9 vs. hPS1WT	0. 8354
	hPS1D9 vs. hPS1E280A	0. 8354
LC3B-I	hPS1WT vs. Mock	0.3879
	hPS1E280A vs. Mock	0.6336
	hPS1D9 vs. Mock	0.3196
	hPS1E280A vs. hPS1WT	0.8784
	hPS1D9 vs. hPS1WT	0.8784
	hPS1D9 vs. hPS1E280A	0. 8784
LC3B-II	hPS1WT vs. Mock	0.2075
	hPS1E280A vs. Mock	*< 0.0001*
	hPS1D9 vs. Mock	0.4852
	hPS1E280A vs. hPS1WT	*< 0.0001*
	hPS1D9 vs. hPS1WT	0.7203
	hPS1D9 vs. hPS1E280A	*< 0.0001*
LC3B II / I	hPS1WT vs. Mock	0.9313
	hPS1E280A vs. Mock	*< 0.0001*
	hPS1D9 vs. Mock	0.9356
	hPS1E280A vs. hPS1WT	*< 0.0001*
	hPS1D9 vs. hPS1WT	0.9356
	hPS1D9 vs. hPS1E280A	*< 0.0001*
MTCH1	*hPS1WT vs. Mock*	*0.0105*
	hPS1E280A vs. Mock	0.0968
	hPS1D9 vs. Mock	0.0684
	hPS1E280A vs. hPS1WT	0.6873
	hPS1D9 vs. hPS1WT	0.6950
	hPS1D9 vs. hPS1E280A	0.8139
Cyclophillin D	hPS1WT vs. Mock	0.8095
	hPS1E280A vs. Mock	*0.0002*
	hPS1D9 vs. Mock	*0.0064*
	hPS1E280A vs. hPS1WT	*0.0003*
	hPS1D9 vs. hPS1WT	*0.0096*
	hPS1D9 vs. hPS1E280A	0.3154
Synthase C	hPS1WT vs. Mock	0.1632
	hPS1E280A vs. Mock	0.0785
	hPS1D9 vs. Mock	*0.0004*
	hPS1E280A vs. hPS1WT	0.5754
	hPS1D9 vs. hPS1WT	0.0751
	hPS1D9 vs. hPS1E280A	0.1631

Table 3

Reagents		
**Name**	**Company**	**Mode of action**
Tunicamycin	Sigma-Aldrich	Induces ER stress by blocking the formation of N-glycosidic linkages, causes G1 arrest
Calcimycin	Sigma-Aldrich	Calcium inophore, uncoupler of oxidative phosphorylation and inhibitor of mitochondrial ATPase activity
Antimycin A	Sigma-Aldrich	Inhibitor of electron transfer at complex III. Induces apoptosis
DMSO	Sigma-Aldrich	
Synthetic human Aβ 1-42	GenicBio	Aggregation and oligomerization leading to toxicity
DAPT	Sigma-Aldrich	Inhibitor of γ-secretase, blocks Notch signaling
2-APB	Sigma-Aldrich	IP_3 receptor antagonist. TRP channel modulator, stimulates store-operated calcium (SOC) release at low concentrations (< 10 μM) and inhibits it at higher concentrations (>50 μM), Modulator of TRP channels.
Bradykinin	Sigma-Aldrich	Bradykinin receptor agonist: stimulates intracellular calcium activity, GPCR activation, IP3-dependent Ca^{2+} ER release
Calcein acetoxymethyl ester	Sigma-Aldrich	Cell-permeable dye, fluorescence of calcein is quenched by Co^{2+} in the cytosol but not in mitochondria
Cyclosporin A	Sigma-Aldrich	Calcineurin inhibitor
Ionomycin	Sigma-Aldrich	Calcium ionophore
FCCP	Sigma-Aldrich	Oxidative phosphorylation uncoupler
Compound W	Tocris Bioscience	Inhibitor of γ-secretase; causes a decrease in the released levels of Aβ42 and notch-1 Aβ-like peptide 25 (Nβ25).

Table 4

Antibodies			
**Name**	**Company and serial number**	**Weight (kDa)**	**Source**
BiP	BD-610979	78	Mouse
Caspase3c	Cell signaling 9661	17-19	Rabbit
CHOP	Cell signaling 2895	27	Mouse
Cyclophilin D	Millipore AP-1035	17	Mouse
GADD 34	ProteinTech 10449-1-AP	100	Rabbit
LC3B	Cell signaling 2275	14-16	Rabbit
MTCH1	LSBio C409602	32	Rabbit
PDI	Biovision 5601	57	Rabbit
PINK1	Abcam 23707	50-60	Rabbit
RIPK1	BD-610458	32-74	Mouse
pRIPK3	Abcam 195117	55	Mouse
Synthase C	Abcam 181243	8-14	Rabbit
Lamp1	SantaCruz sc-20011	120	Rabbit
PS1	Cell signaling 3622	55-22	Rabbit
PS1 N-terminal	Millipore 1563	55-35	Rat
PS1	Abcam 15456	28	Mouse
APP CT-20 (CTL, FL)	Millipore 171610	110	Rabbit
GAPDH	Millipore MAB374	36	Mouse
KDEL	PA1-013 Thermo Scientific	57-78-94	Rabbit
Tom20	SantaCruz sc-11415	20	Rabbit
GM130	Abcam 52649	112	Rabbit
Lamp1	14-107182 Thermo Scientific	120	Rat

Figure S1

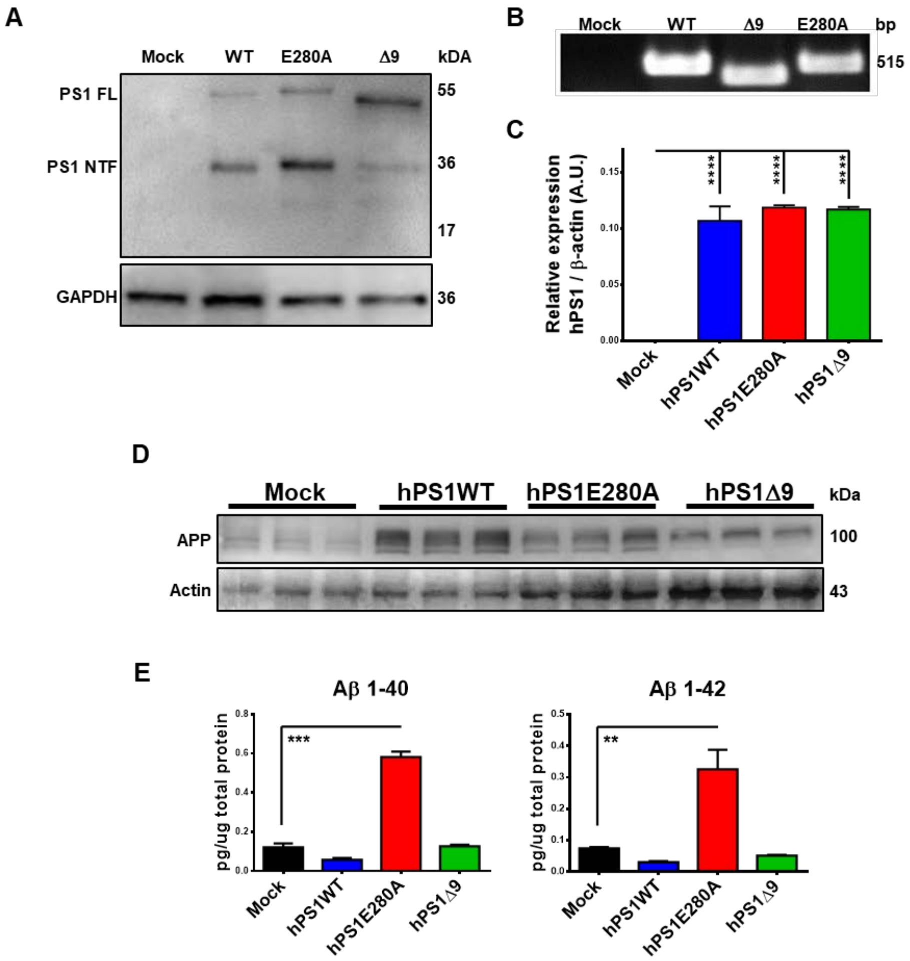

Figure S1. (A) Western blot for PS1 levels in stably transfected N2A cells assessed using a human PS1 specific antibody (Millipore MAB1563). Specific expression of human PS1 is visible in all stably transfected cell lines. (B) Agarose gel electrophoresis for PCR products using human PS1 primers in stably transfected N2A cells. (C) Human PS1 expression in stably transfected N2A cells tested by qPCR. (D) Western blot for APP levels in mock and stably transfected N2A cells. (E) ELISA assays for Murine Aβ 1-40 and 1-42 in stably transfected N2a cells. Only hPS1E280A cells show significantly increased levels when compared to mock cells. $^{**}P < 0.01$, $^{***}P < 0.001$. Data are mean ± SEM, One-Way ANOVA.

Figure S2

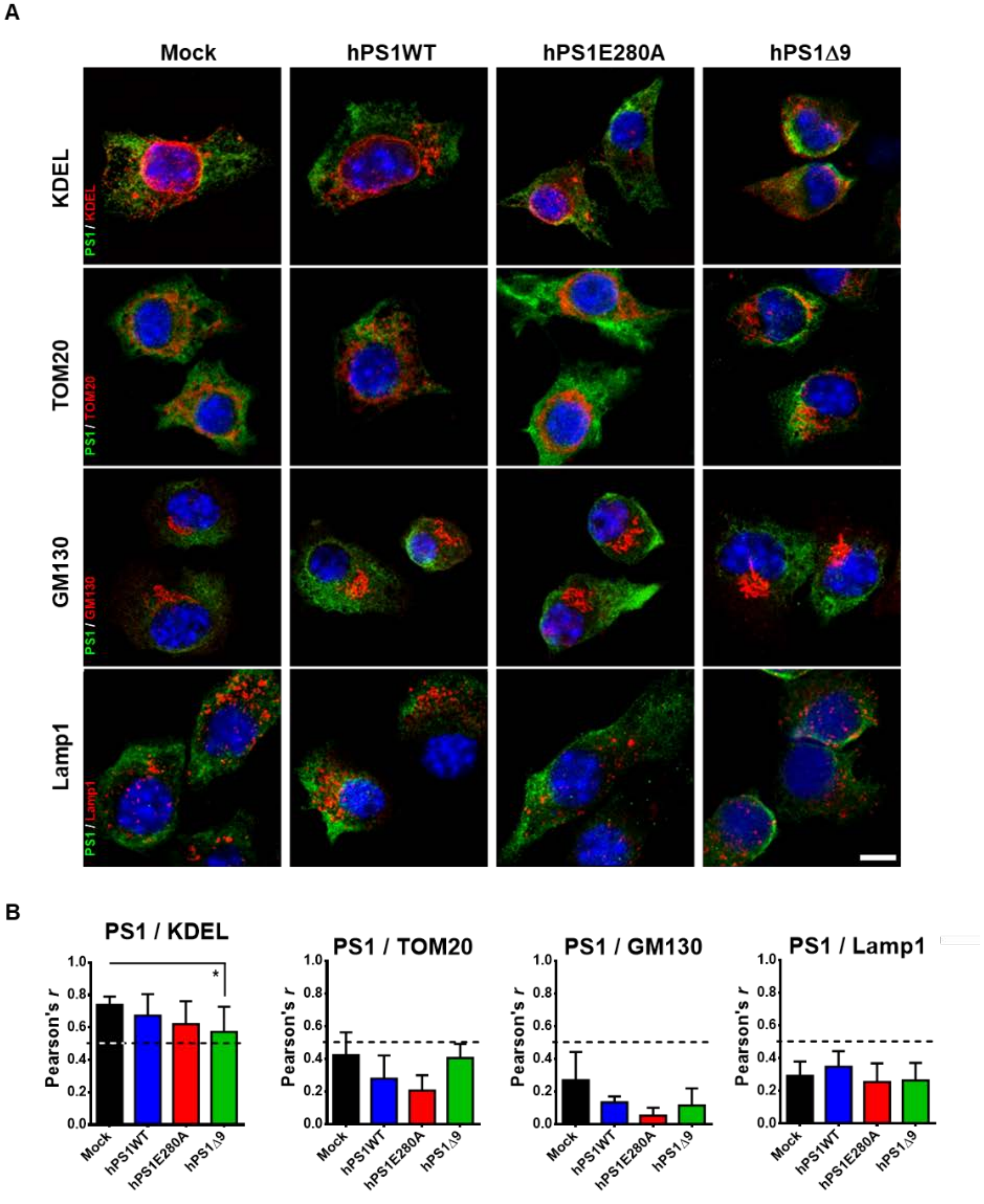

Figure S2. (A Representative confocal microscopy pictures of Mock, hPS1WT, hPS1E280A and hPS1Δ9 N2a cells assessing PS1 (red) colocalization with markers (in green) for ER (KDEL), mitochondria (Tom20), Golgi (GM130) and lysosomes (Lamp1).(B) Signal was quantified in 3 independent experiments in at least P8 cells per experiment and cell line. Pearson's coefficients showed significant differences for PS1-KDEL colocalization between mock and hPS1Δ9 N2a cells. Dotted lines represent colocalization thresholds. *P < 0.05. Data are mean ± SEM, Two-Way ANOVA. Bar=7 μm

Figure S3

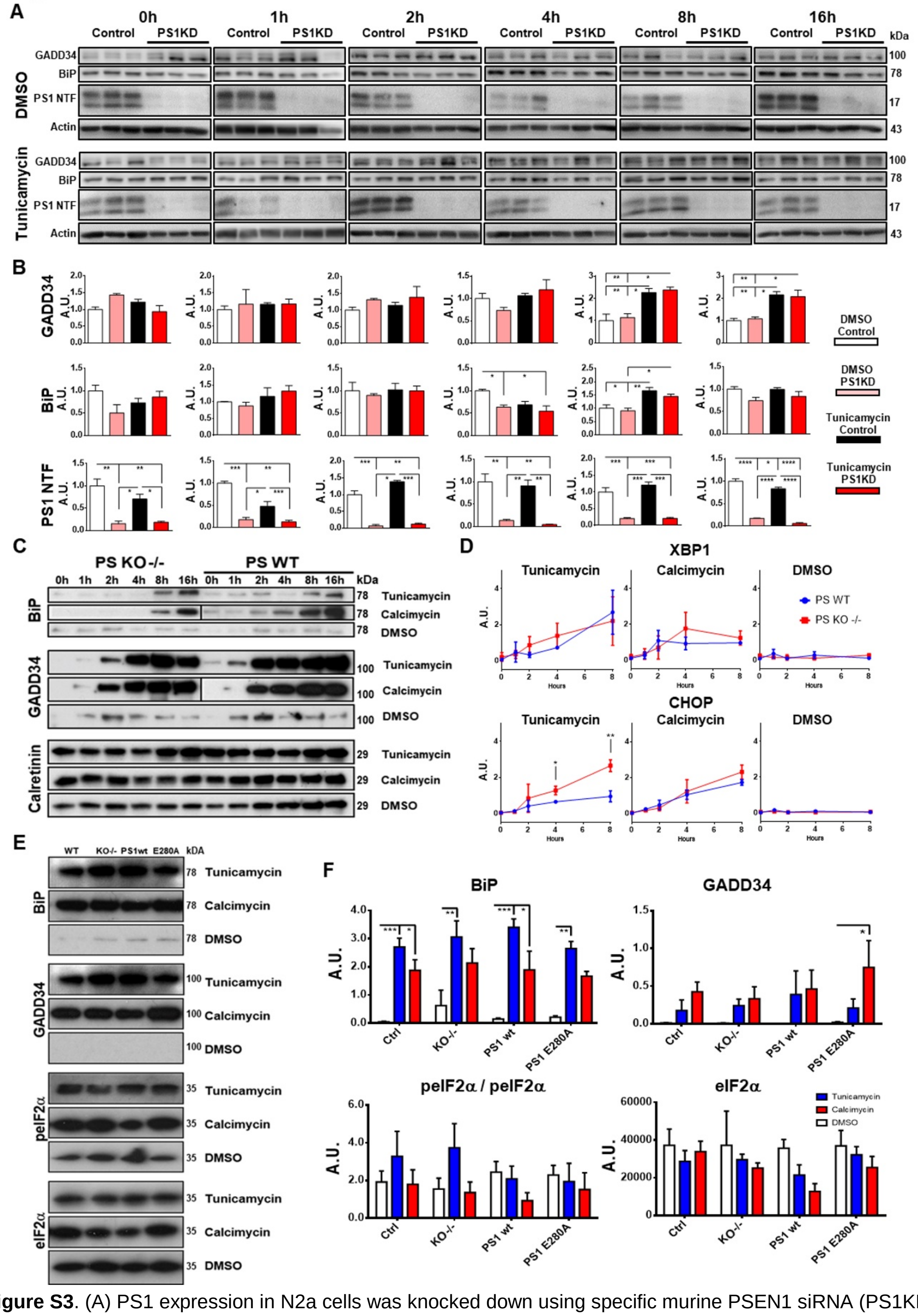

Figure S3. (A) PS1 expression in N2a cells was knocked down using specific murine PSEN1 siRNA (PS1KD). PS1KD cells and their controls were treated with Tunicamycin to induce ER stress at different time points using BiP, Gadd34 as markers and actin as loading control. (B) Densitometric analysis of BiP, Gadd34 and PS1 shows increased levels of GADD34 and BiP at 8 h, and of GADD34 at 16 h in treated cells, independently on PS1 levels. *P < 0.05, **P < 0.01, ***P < 0.001, ****P < 0.0001. Data are mean ± SEM, Two-Way ANOVA. (C) PS1/PS2 knockout MEF cells (PS KO -/-) and expressing PS1 WT were also treated with Tunicamycin and Calcimycin to induce ER stress at different time points using BiP, GADD34 as markers and calretinin as loading control. Samples in the Calcimycin blots for BiP and GADD34 were loaded following a different order. For this panel there were cropped and reordered following the general loading order. (D) Expression of UPR markers CHOP and XBP1 was evaluated with qPCR. (E) ER stress induction assessment with BiP, GADD34, peIF2α and eIF2α levels, after 8 h of Tunicamycyn or Calcymicin treatments in PS1 stably rescued MEFS. (F) Densitometric analysis of BiP, GADD34, peIF2α and eIF2α. Tunicamycin increased BiP levels in all cell lines. There were no differences between PS KO-/- and PS1 rescued cells. *P < 0.05, **P < 0.01, ***P < 0.001. Data are mean ± SEM, Two-Way ANOVA.

Figure S4

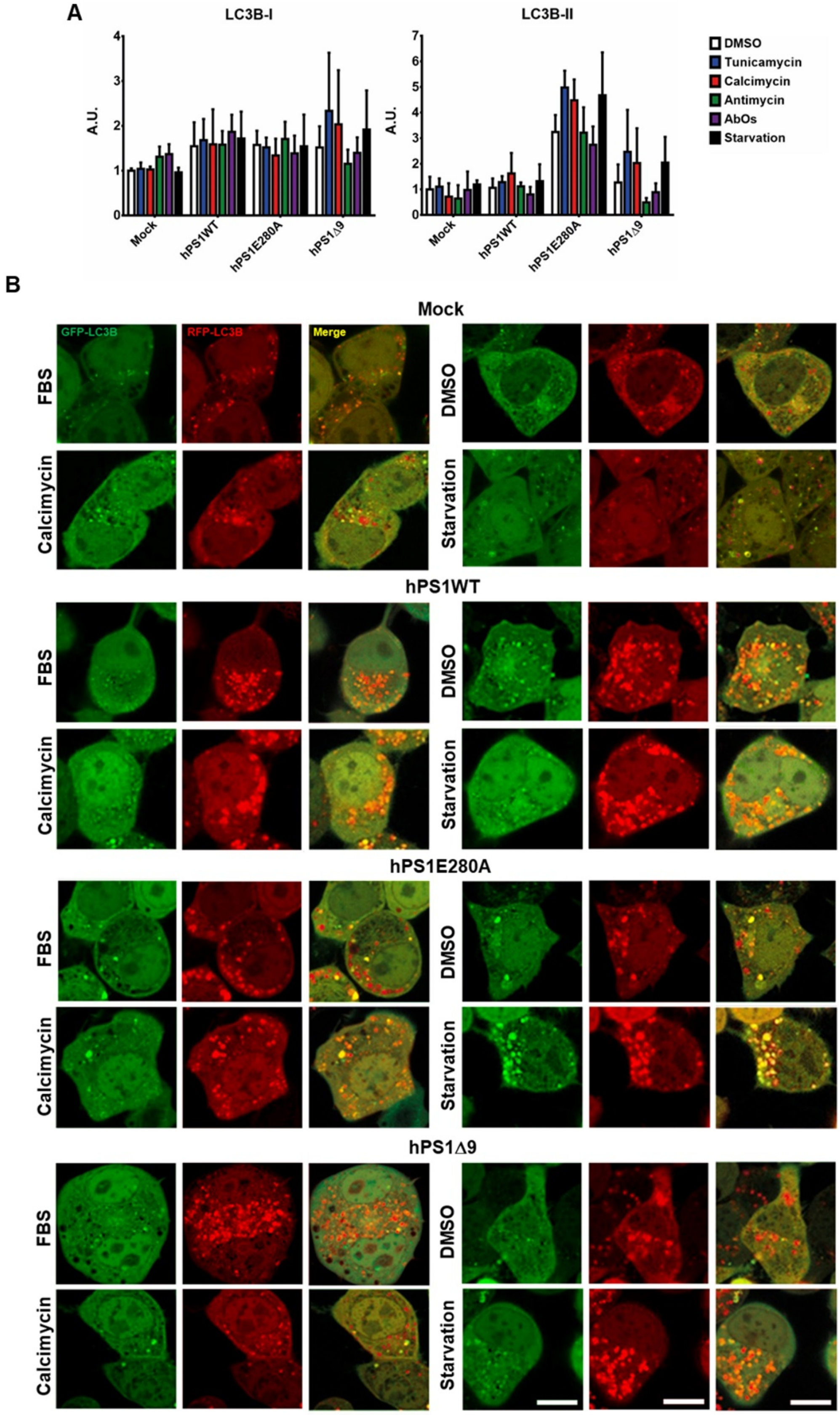

Figure S4. (A) LC3B I and LC3B II densitometric quantification normalized to PDI and compared to basal conditions. (B) Green (GFP-LC3B) channel, red (RFP- LC3B) channel and merged images observed in panel 2C. Transfected cells were treated with vehicle (DMSO) and stressors (Calcimycin and Starvation). The transition from autophagosomes (yellow) to autolysosomes (red) can be visualized by the specific loss of GFP fluorescence upon acidification of the autophagosome following lysosomal fusion. Bar = 7μm.

Figure S5

A

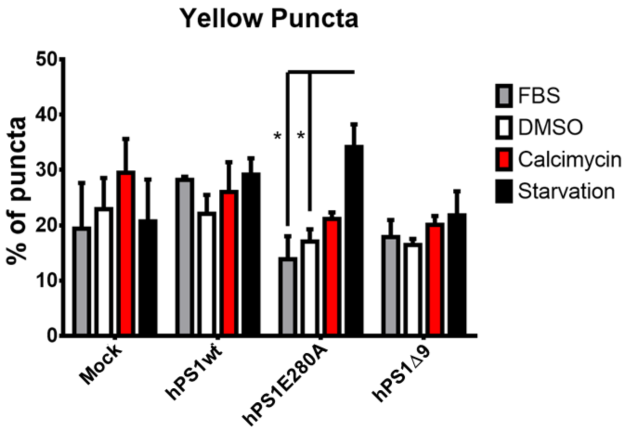

B

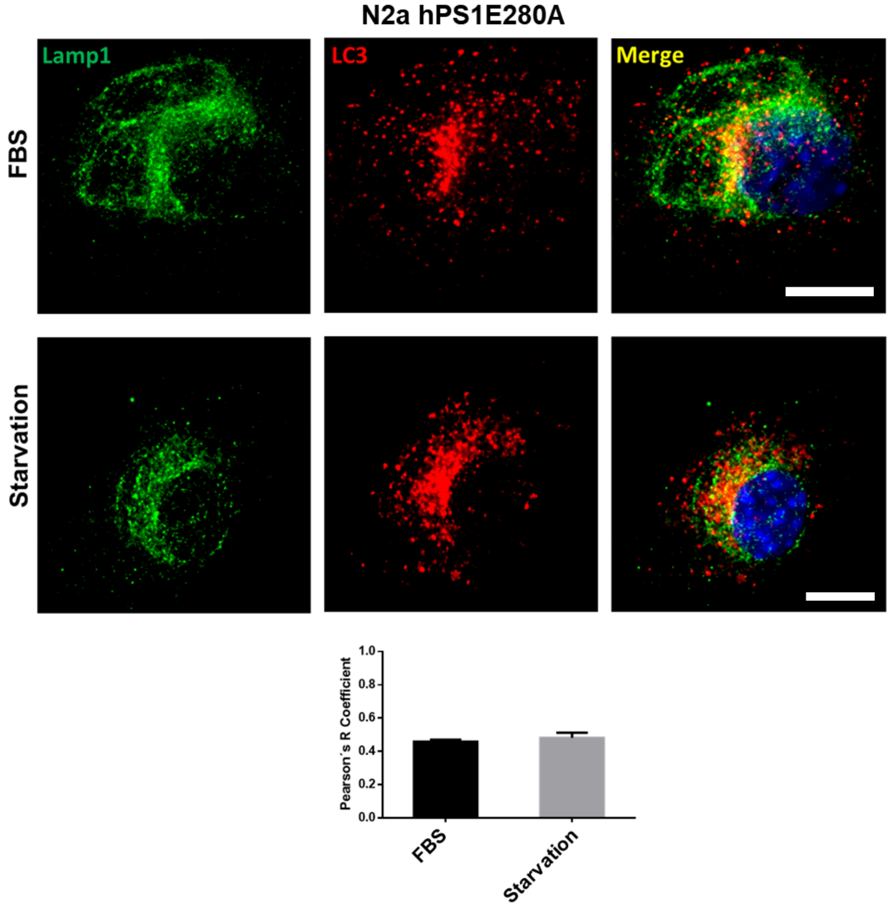

Figure S5. (A). Yellow puncta quatification in N2a cells transfected with the RFP-GFP-LC3B construct. Transfected cells were treated with vehicle (DMSO) and stressors (Calcimycin and Starvation) for 16 h. (B). Representative confocal microscopy pictures of PS1E280A N2A starved for 16 h and their FBS controls. Localization and signal of LC3B (red) and Lamp1 (green) was quantified in 3 independent experiments. Pearson's coefficients showed no significant differences between starved and non-starved cells. Bar= 7μm *P < 0.05, **P < 0.01, ***P < 0.001. Data are mean ± SEM, Two-Way ANOVA for A and T-test for B.

Figure S6

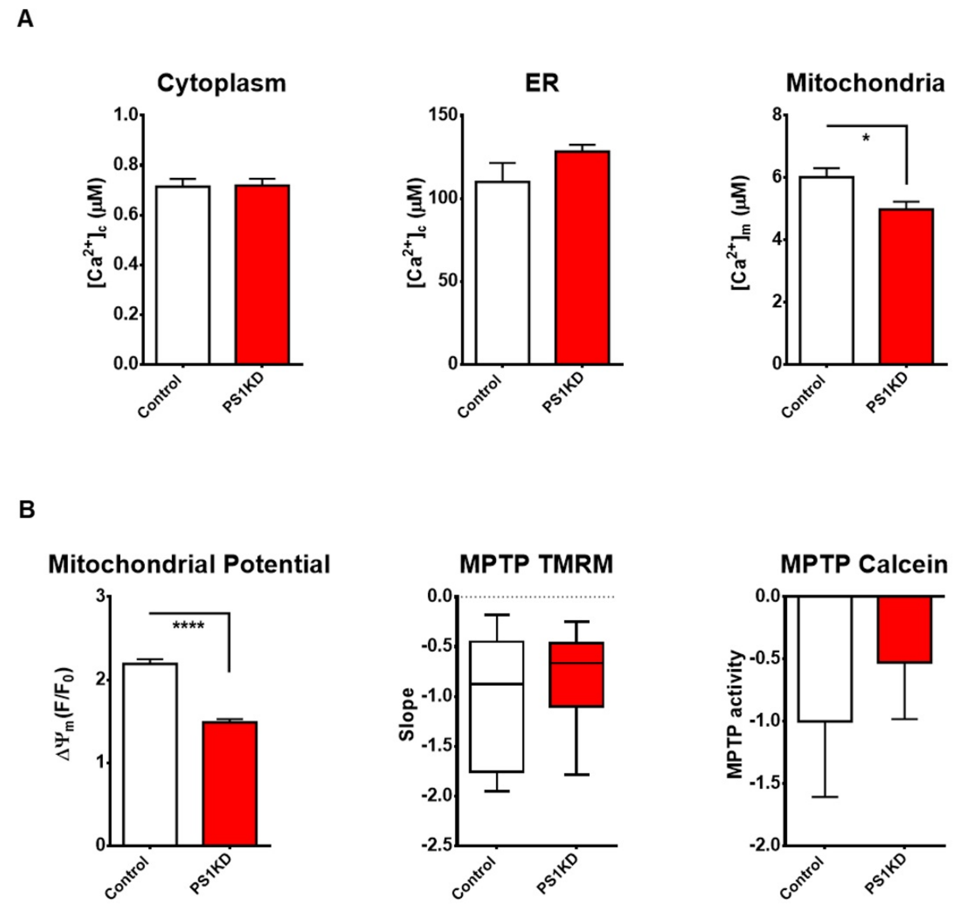

Figure S6. N2a PS1KD were used to measure intracellular calcium (Ca2+) concentrations in basal conditions using transient transfection of a compartment-specific aequorin constructs. (A) Bar graphs of the maximum cytosolic, ER and mitochondria calcium in PS1KD and control cells. Only mitochondria calcium showed decreased levels in PS1KD cells. (B) Bar graphs representing TMRM intensity as measurement of mitochondrial membrane potential ($\Delta\Psi_m$) and MPTP activity during live imaging. Only $\Delta\Psi_m$ showed significantly decreased levels in PS1KD cells, while both approaches for MPTP measurement showed no differences. Mean and ±SEM are presented for all experiments, Two Way ANOVA, *p<0.05, ****p<;0.0001 n>30 measurements.

Figure S7

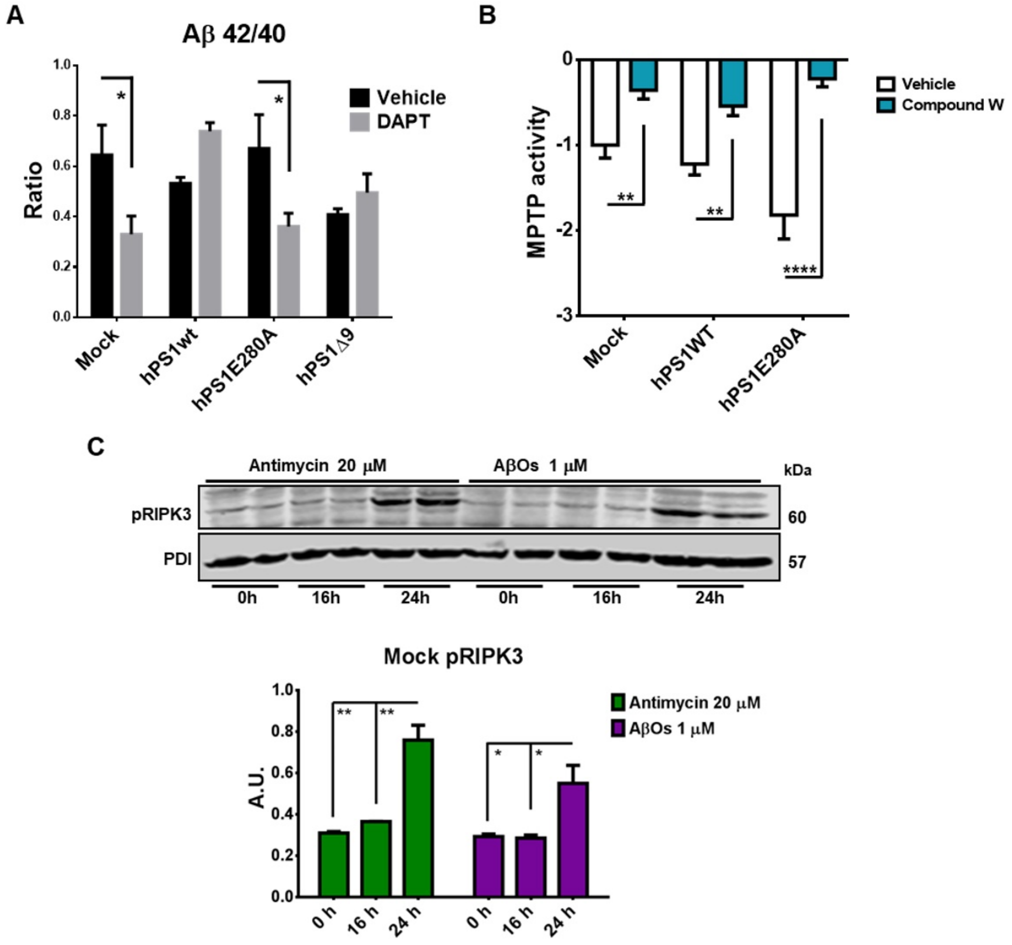

Figure S7. (A) Aβ 1-42/1-40 ratio calculated from peptide levels in mock, hPS1WT and hPS1E280A N2a cells using ELISA assays after 16 h of treatment with 250 μM of DAPT. DAPT treatment significantly decreased ratios in mock and hPS1E280A cells. *P < 0.05, data are mean ± SEM, Two-Way ANOVA. (B) MPTP activity in mock, hPS1WT and hPS1E280A N2a cells after 48h of treatment with 10 mM of Compound W, a γ-secretase inhibitor. Compound W significantly decreased MPTP activity in all cell lines. **P < 0.01, ****P < 0.0001, data are mean ± SEM, Two-Way ANOVA. (C) Representative western blot and densitometric analysis for pRIPK3 and PDI as loading control of control N2a cells treated with antimycin 20 mM and 1-42 Ab oligomers 1 mM at 0, 16 and 24 h. pRIPK3 levels are significantly higher for both treatments after 24 h. *P < 0.05, **P < 0.01, data are mean ± SEM, Two-Way ANOVA.
